# Hyaluronic Acid Microplates for Intra-articular Lubrication and Cartilage Protection in Post-traumatic Osteoarthritis

**DOI:** 10.1021/acsami.5c11890

**Published:** 2025-10-12

**Authors:** Agnese Fragassi, Antonietta Greco, Megan Keech, Amelia Soltes, Fang Yu, Sayanti Brahmachari, Roberto Palomba, Martina di Francesco, Miguel Echanove Gonzalez De Anleo, Froilan Granero-Molto, Luca Ceseracciu, Veronica Papa, Luca Goldoni, Aiman Abu Ammar, Richard D’Arcy, Haytam Kasem, Craig Duvall, Paolo Decuzzi

**Affiliations:** † Laboratory of Nanotechnology for Precision Medicine, 121451 Fondazione Istituto Italiano di Tecnologia, Via Morego 30, Genova 16163, Italy; ‡ Department of Biomedical Engineering, 5718 Vanderbilt University, 2301 Vanderbilt Place, Nashville, Tennessee 37235-1631, United States; § Cell Therapy Area, 16755 Clínica Universidad de Navarra, Av. de Pío XII, 36, Pamplona, Navarra 31008, Spain; ∥ Materials Characterization Facility, Istituto Italiano di Tecnologia, Via Morego 30, Genova 16163, Italy; ⊥ Biotribology Inter-Disciplinary Research Center, 72258 Azrieli College of Engineering Jerusalem, 26 Yaakov Shreibom Street, Ramat Beit Hakerem, Jerusalem 9103501, Israel; # Department of Pharmaceutical Engineering, 72258 Azrieli College of Engineering Jerusalem, Jerusalem 9103501, Israel; ∇ Department of Medicine, Division of Oncology, Stanford University School of Medicine, 269 Campus Drive, Stanford, California 94305, United States; ○ Department of Pharmaceutical and Pharmacological Sciences, University of Padova, Via F. Marzolo 5, Padova 35131, Italy; ◆ Department of Medicine and Surgery, nanomedicine Center (NANOMIB), University of Milano-Bicocca, Via Follereau 3, Vedano Al Lambro 20854, Italy; ¶ Chemical Engineering, School of Engineering of Matter, Transport and Energy, Arizona State University, Tempe, Arizona 85287, United States

**Keywords:** osteoarthritis, hyaluronic acid, microparticles, lubrication, anti-inflammatory activity

## Abstract

Osteoarthritis (OA) is the most common joint disorder, characterized by a vicious cycle of synovial inflammation and cartilage degradation. Intra-articular injection of hyaluronic acid (HA)-based products, one of the currently available treatments, provides only temporary symptomatic relief without addressing the underlying inflammation. Here, we engineered several configurations of 20 × 5 μm square-shaped HA-based hydrogel microparticles (μHA) by photopolymerizing HA–methacrylate chains within a sacrificial template. The μHA mechano-pharmacological properties were tuned by adjusting the HA concentration, molecular weight, and degree of methacrylation, resulting in microparticles with a Young’s modulus ranging from a few tens (30 kPa) to a few hundred (200 kPa) kilopascals; a structure stable for over a month under oxidative stress conditions; and reduced friction in simulated synovial fluids. Under H_2_O_2_-induced oxidative conditions, μHA decreased the production of proinflammatory cytokines (IL-6, IL-1β, and TNF-α) in human chondrocytes to basal levels. In a three-dimensional OA cartilage model, μHA reduced glycosaminoglycan release and matrix metalloproteinase-13 activity, demonstrating chondroprotective effects. In a rigorous murine model of early-stage post-traumatic OA, a single intra-articular injection of μHA lowered proinflammatory gene expression in the synovium to basal levels. In summary, μHA offers a drug-free approach to managing OA by enhancing lubrication and reducing inflammation, providing a sustained therapeutic activity over several weeks.

## Graphical abstract

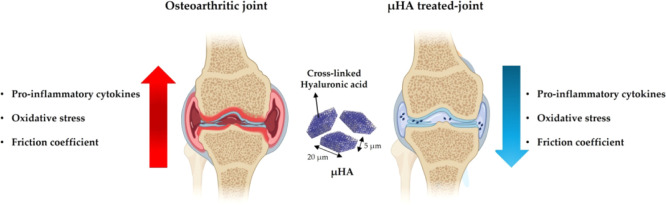

## Introduction

Osteoarthritis (OA) is the most prevalent joint disorder, characterized by the progressive degradation of articular cartilage, increased synovial inflammation, and subchondral bone sclerosis, ultimately resulting in chronic pain and significant functional impairment.^1–3^ Globally, OA affects nearly 600 million individuals (8% of the population), with a higher incidence among the elderly and women.^4^

OA is classified as primary, arising from unknown causes, or as post-traumatic osteoarthritis (PTOA), secondary to injury. PTOA is triggered by joint mechanical injury (e.g., anterior cruciate ligament or meniscal tears, intra-articular fractures, patellar dislocation) and therefore often affects younger and highly active individuals. In addition to direct cartilage and osteochondral damage, precipitating trauma typically induces acute hemarthrosis and a sharp rise in inflammatory mediators (e.g., IL-1β, TNF-α), matrix-degrading enzymes, and damage-associated molecular patterns within the synovial fluid, setting in motion catabolic cascades that can persist beyond the initial event. Malalignment, residual instability, meniscal deficiency, and altered joint loading further accelerate disease progression in the months to years following injury,^5^ imposing a substantial and earlier-than-usual burden of pain, disability, and productivity loss on patients, as well as a significant economic burden on healthcare systems and society at large.^6^ Despite this, therapeutic options for OA/PTOA remain limited. Current standard treatments primarily rely on the intra-articular injections of various agents, which can only transiently alleviate pain and modulate inflammation.^7^ At present, no clinically available therapies can reverse cartilage damage and restore the original joint function. Although the pathogenic mechanisms driving OA and PTOA are still not fully understood, the most widely accepted hypothesis is that a significant factor contributing to the onset and progression of this disease is a substantial decline in joint lubrication.^8^ This decline leads to tissue damage, resulting in the shedding of cartilage fragments into the synovium, which then triggers the inflammation of synovial cells. The soluble mediators released by these inflamed cells act on exposed chondrocytes, prompting them to secrete factors, such as metalloproteinases, in an attempt to remodel the matrix and protect themselves. However, this remodeling inevitably causes further cartilage degradation, initiating and perpetuating a vicious cycle involving multiple cells and compartments within the entire joint.^1,9^ Under physiological conditions, cartilage lubrication relies on key macromolecules in the synovial fluid, including hyaluronic acid (HA), lubricin, and phospholipids, which synergistically work to minimize friction between the articulating cartilage surfaces.^10–12^ HA, in particular, serves as the structural backbone of the lubrication layer within the synovial fluid while exhibiting unique anti-inflammatory biochemical functions.^13–16^ Studies in various OA animal models and in patients have demonstrated a correlation between cartilage disruption and a progressive reduction in HA concentration and molecular weight within the synovial fluid.^17,18^ These alterations are partially driven by reactive oxygen species (ROS) released by inflamed chondrocytes and synovial cells, further perpetuating the vicious cycle described above.^19,20^

We seek to create a longer-lasting HA-based formulation that could restore joint lubrication to alleviate and potentially reverse OA progression. Indeed, the intra-articular injection of HA, known as viscosupplementation, is a well-established therapeutic strategy for OA.^7,21^ Clinically approved exogenous HA products include those with relatively low molecular weight (HYALGAN, 500–730 kDa); intermediate molecular weight (Orthovisc, 1000–2900 kDa), though still lower than that of healthy synovial fluid; and cross-linked hyaluronan with high molecular weight (Synvisc, 6000 kDa). Cross-linking aims to increase the intra-articular dwelling time and delay the reduction in molecular weight of the HA chains, thereby prolonging the effect of the intervention.^22^ These products form hydrogels due to the network of randomly entangled chains; these physical gels can pose challenges during intra-articular administration due to their nonuniform size and shape, requiring relatively high injection forces. Various nanoparticles, made from different materials and with diverse surface modifications, have been proposed as an alternative strategy due to their small size, colloidal stability, easier injection, and potential to also be utilized for drug delivery.^23–25^ Unfortunately, nanoparticles are rapidly cleared from the joint within a few days due to the inflamed, hyperpermeable synovium, thus necessitating frequent intra-articular injections. Microscopic particles, on the other hand, have demonstrated longer intra-articular retention times and sustained delivery of both small molecules as well as nanomedicines.^26–28^ For instance, Ratclifee and colleagues demonstrated that albumin microspheres were cleared slowly, with no significant difference between normal and inflamed joints.^29^ In a systematic analysis, the group of Allemann studied the intra-articular fate of fluorescent poly­(d,l)-lactide particles of different sizes, observing that 300 nm particles leaked from the joint regardless of the inflammatory status; 3 μm particles were retained only in the noninflamed joint; while complete retention, independently of the inflammatory status, was documented only for 10 μm particles.^30^ Along the same lines, in a murine post-traumatic OA model, the authors demonstrated that encapsulating nanoparticles carrying siRNA against MMP-13 in poly­(lactic-*co*-glycolic acid) (PLGA) microparticles induced prolonged gene expression knockdown and reduced MMP-13 protein production over a 28-day study. This effect was not observed when siRNA nanoparticles were freely injected intra-articularly.^31^

In the current work, we developed HA-based microparticles designed to enhance intra-articular lubrication and protect cartilage against wear. To achieve this, HA chains of varying molecular weights were chemically modified into photopolymerizable HA–methacrylate (HA-MA) prepolymers with different degrees of methacrylation. These prepolymers were subsequently assembled into thin, square-shaped microscopic hydrogel particles, named HA microplates (μHA). This was accomplished using a top-down approach that combined a sacrificial template strategy with the photopolymerization of HA-MA. The HA microplates were extensively characterized for their physicochemical, mechanical, and tribological properties. Additionally, preliminary studies were performed to assess biocompatibility and therapeutic activity in a preclinical model of post-traumatic OA.

## Materials and Methods

### Materials

Polydimethylsiloxane (PDMS) (Sylgard 184) and elastomer were purchased from Dow Corning (Midland, Michigan, USA). Hyaluronic acid (10, 50, and 500 kDa) was obtained from Creative Pegworks (Durham, North Carolina, USA). Poly­(vinyl alcohol) (PVA, MW 31 000–50 000), poly­(d,l-lactide-*co*-glycolide), glycidyl methacrylate, lithium phenyl­(2,4,6-trimethylbenzoyl)­phosphinate (LAP), hydrogen peroxide (H_2_O_2_) 30% (w/w) in H_2_O, albumin–fluorescein isothiocyanate conjugate, *N*-ethyl-*N*′-(3-(dimethylamino)­propyl)­carbodiimide, *N*-hydroxysuccinimide, ATDC5 cell line, MTT assay l-ascorbic acid, dexamethasone, and Quant-iT PicoGreen dsDNA Assay Kit were purchased from Sigma-Aldrich (Saint Louis, Missouri, USA). High-glucose Dulbecco’s modified Eagle’s minimal essential medium (DMEM)/F-12 GlutaMAX, high-glucose Dulbecco’s modified Eagle’s minimal essential medium (DMEM) penicillin, streptomycin, and heat-inactivated fetal bovine serum (FBS) were purchased from Gibco (Invitrogen Corporation, San Giuliano Milanese, Milan, Italy). Poly-d-lysine was purchased from Gibco–Thermo Fisher Scientific (Waltham, Massachusetts, USA). Synovial fluid concentrate was purchased from Limbs & Things (Bristol, UK). A rigid hard counterface made of 76 mm × 26 mm × 1 mm soda lime glass plates was purchased from Paul Marienfeld GmbH & Co. KG (Germany). Human chondrocytes and chondrocyte medium were purchased from Innoprot (Bizkaia, Spain). Human TNF-α (Tumor Necrosis Factor Alpha), Interleukin-6, and Interleukin-1β enzyme-linked immunosorbent assay (ELISA) kits were obtained from Twin Helix SRL (Milan, Italy). CellCarrier Spheroid ULA 96-well microplates were obtained from Revvity (Waltham, Massachusetts, USA). MEM-α, penicillin, streptomycin, heat-inactivated fetal bovine serum (FBS), Corning ITS+ Premix Universal Culture Supplement, mouse study SYBR primers and reagents, as directed by standard protocols, were purchased from Integrated DNA Technologies (Coralville, Iowa, USA) and ABclonal (Woburn, Massachusetts, USA), respectively. C57BL/6 mice were purchased from Jackson Laboratory (Bar Harbor, Maine, USA). Recombinant human TGF-beta 3 protein was obtained from R&D Systems (Minneapolis, Minnesota, USA), and mouse recombinant IL-1β was purchased from Stemcell Technologies (Vancouver, Canada). Glycosaminoglycans Assay Kit and Fluorimetric MMP-13 Activity Assay Kit were purchased from Fisher Scientific (Pittsburgh, Pennsylvania, USA). C57BL/6 mice were purchased from Jackson Laboratory (Bar Harbor, Maine, USA). HYALGAN (20 mg/2 mL) was purchased from Vanderbilt Medical Center’s Pharmacy for Research Drugs.

### Synthesis and ^1^H NMR Characterization of Hyaluronic Acid–Methacrylate (HA-MA) Prepolymers

Methacrylate (MA) groups were introduced into the HA chains to generate photopolymerizable HA-MA prepolymers through a reaction with glycidyl methacrylate (GM). Specifically, HA-MA polymers with different degrees of methacrylation (DM), ranging from 10 to 30%, were obtained by treating a 0.3% w/v solution of HA (10, 50, 500 kDa) in phosphate buffer (PBS 1×, 40 mL) and dimethylformamide (DMF, 40 mL) with a 50- or 100-fold molar excess of GM in the presence of excess trimethylamine (TEA).^30^ After 12 h, 2 days, or 5 days of stirring at room temperature, the product was purified by precipitation from an excess of acetone, washed twice with an excess of methanol, and lyophilized to obtain the purified product.

NMR characterizations were performed at 298 K on a Bruker Avance 400 MHz spectrometer equipped with a TBO probe and Z-gradients. 128 transients were accumulated after the 90° automatic optimization by using 65 536-digit points, a relaxation delay of 30 s, over a spectral width of 20.49 ppm, with the offset at 6.175 ppm. Deuterium oxide (D_2_O) was used as the solvent, and the polymer concentration was 0.25% by mass fraction.

### Preparation and Characterization of Hyaluronic Acid–Methacrylate (HA-MA) Microplates (μHA)

HA hydrogel microparticles (μHA) appeared as right prisms with a square base of 20 μm × 20 μm and a height of 5 μm and were named hyaluronic acid microplates (μHA). These were fabricated by performing a photopolymerization reaction on the HA-MA prepolymers within a sacrificial template made of polyvinyl alcohol (PVA). The PVA template was realized using a soft lithography approach, as previously described by the authors.^31,32^ Specifically, the PVA sacrificial template was obtained following a sequence of replica-molding steps. First, a silicon master template was fabricated using direct laser writing; this silicon template presented an array of square-based wells with an edge length of 20 μm and a depth of 5 μm and a separation distance of 20 μm between adjacent wells. The silicon template was then replicated into an intermediate poly­(dimethylsiloxane) (PDMS) template. This was obtained by covering the master template with a PDMS:curing agent mixture in a 10:1 ratio and cured in an oven at 60 °C for 4 h. Subsequently, the PDMS template was peeled off the master template and replicated into a PVA template. The PVA template was produced by pouring a 10% w/v PVA solution onto the PDMS template and allowing all the water to evaporate at 60 °C. The PVA sacrificial template is an identical replica of the original master template and, as such, features an array of square-based wells with an edge length of 20 μm and a depth of 5 μm.

The fabrication of μHA involved, first, the dissolution of HA-MA precursors in a water–glycerol (W/G) solution containing 0.1% (w/v) lithium phenyl-2,4,6-trimethylbenzoylphosphinate (LAP). The high-molecular-weight (MW) HA-MA prepolymers were dissolved in a W/G solution with a glycerol concentration of 30%, while the low-molecular-weight HA-MA prepolymers (10 kDa) were dissolved in a W/G solution with a glycerol content of 40%. LAP, prepared as a stock solution in water (30 mg/mL), was added to the aqueous phase of the HA-MA W/G solutions. The resulting aqueous solutions were spread over the wells of the PVA template and cross-linked with UV light using a multistep polymerization strategy (40 μL per template). In brief, 10 μL of the HA-MA W/G solution was spread on the PVA template, then the loaded template was immediately exposed to UV light for 5 min, initiating the polymerization reaction. This process was repeated three more times to load the entire volume. Subsequently, the PVA template was dissolved in 25 mL of water under magnetic stirring for 2 h to facilitate the dissolution of the PVA and the release of μHA. These were purified from the PVA solution using polycarbonate membrane filters (40 μm) and collected through two sequential centrifugations (2500 *g* for 5 min). To assess the influence of the HA-MA concentration on the resulting microparticles, the fabrication process was carried out using HA-MA W/G solutions at three different concentrations (5, 10, and 15% w/v) within the gel formation conditions corresponding to the specific W/G ratio.

### Morphological and Mechanical Characterization of μHA

All μHA formulations were characterized via Multisizer 4 COULTER particle counter (Beckman Coulter, California, USA) to examine size distribution and via scanning electron microscopy (SEM, Elios Nanolab 650, FEI) to assess the actual nonspherical shape. SEM analyses were performed on samples dried overnight at room temperature or samples dehydrated with ethanol. Specifically, samples were dehydrated at increasing concentrations of ethanol in water solutions (from 30 to 100%). Ethanol dehydration was followed by replacement with hexamethyldisilazane, which was allowed to evaporate in a fume hood overnight. In addition, a custom protocol was used to fluorescently stain μHA: 120 000 μHA particles were resuspended in 160 μL of PBS and 80 μL of 1 mg/mL Alexa Fluor 488 Wheat Germ Agglutinin (WGA) solution was added. Particles were left under agitation for 1 h on a bascule at room temperature. Particles were centrifuged at 500 rpm for 5 min at 4 °C. After the supernatant was removed, particles were washed twice in 1 mL of DI water. After the final wash, 1 mL of PBS was added, and 15 μL of the suspension was spread on a cover glass and allowed to dry for 1 h under the chemical hood. Particles were observed under the confocal microscope by mounting the cover glass on a microscopic slide. A 63× objective was used, and a z-stack series was acquired with 8 steps of 1000 nm each. Images were realized using an A1-Nikon confocal microscope (Nikon Corporation, Japan). A 3D reconstruction was generated by using NIS-Software (Nikon Corporation, Japan).

The mechanical properties of μHA were investigated by using a Chiaro nanoindenter (Optics 11 Life). The device used a piezo-driven actuator to apply a controlled indentation while evaluating the reaction force from the deflection of a cantilever probe measured by interferometry. The system was equipped with a spherical probe of 8.5 μm diameter and a cantilever with a stiffness of 0.250 N/m. Tests were conducted on the μHA in deionized water inside a glass Petri dish. μHA particles were identified individually through an inverted microscope coupled with the nanoindenter and indented in displacement control, at a rate of 4 μm/s, over the particle core to avoid any border effects. Typical indentations reached a maximum load of 0.15–0.20 μN and a penetration depth of ∼ 400 nm, which is much smaller than the nominal height of the μHA (∼5000 nm). After testing, the load-indentation curves were fitted with the classical Hertz equation to extract the apparent Young’s modulus (*Y*) under compression loading. From the slope of the force–displacement curves, the Young’s modulus was calculated through the classical Hertzian equation *F = 2RYh*, where *F* is the applied force, *h* is the displacement of the tip, and *R* is the radius of the tip. To reduce any possible influence of the substrate on the measurements, the force–displacement curve fitting was limited to the initial loading portion, up to 30% of the maximum load. Only indentations with a fitting coefficient *R* ^2^ >0.9 were selected, and at least 10 microparticles per condition were considered. Additionally, Dynamic Mechanical Analysis (DMA) tests were performed with the same equipment. In DMA, a sinusoidal load oscillation was applied at different frequencies once the maximum load was reached. The phase difference δ between the input (load) and output (deformation) was recorded as a function of the frequency. The tangent of the phase difference angle, denoted as tan δ, was computed, representing the ratio between dissipative and conservative energy during a single oscillation.

### Tribological Characterization of μHA

The tribological behavior of the μHA was investigated using a customized two-axis tribometer designed and constructed in the Biotribology Interdisciplinary Research Center at the Azrieli College of Engineering in Jerusalem.^33^ Based on a moving horizontal counterface, this device enables the investigation of the tribological properties (friction, adhesion, and peeling) of different materials under dry or wet contact conditions, according to need. The instrument included a drive unit and a measurement unit. The drive unit included three translations stages: two motorized stages to move the countersurface vertically and laterally within the working plane for applying loads between the components of the friction pair, and one manual stage to adjust the contact location between the mating surfaces along the third axis. This pair comprised a Teflon upper disk (a cylinder cut from a Teflon rod with dimensions 10 mm height and 10 mm diameter)—the Teflon upper disk was polished to achieve an average arithmetic roughness of 250 nm on its rubbing surface—and a bottom glass counter surface (76 mm × 26 mm × 1 mm). A passive, self-alignment system—based on the principle of two free rotation axes—was utilized to mount the Teflon disk on the tribometer and guarantee a full contact parallelism with the mating countersurface during the friction tests. The measurement unit integrated two high-resolution load cells (FUTEK’s FSH00092-LSB200) to measure force variations (0.1 mN) in both normal and tangential directions. The measurements were sampled with a multifunctional data acquisition board Lab-PC- NI USB-6211 (National Instruments Co., Austin, Texas, USA) and processed using a LabVIEW 2017 software package (National Instruments Corporation, Texas, USA). For each experiment, the glass counterface was cleaned with ethanol. Then, the Teflon rod and glass substrate were mounted on the tribometer, and the glass counterface was covered with 500 μL of simulated synovial fluid, either blank or enriched with μHA. A single friction cycle consisted of 8 consecutive steps: (*i) approaching:* the glass counterface is moved up in the vertical direction and brought into contact with the Teflon rod, leading to a gradual increase in the normal load P until the desired predefined value of 5.8 N is reached; *(ii) waiting dwell time*: the system is left to accommodate for 0.5 s; *(iii) tangential movement*: the glass counterface is moved in the tangential direction at a constant sliding velocity of 1 mm·s^–1^ for a total crossed distance of 20 mm. During this step, the normal load is constant, while the tangential force opposing the sample motion is recorded; (*iv) waiting*: the system is left to accommodate for 0.5 s; *(v) disconnecting*: the glass counterface is withdrawn in the vertical opposite direction until a complete separation of the mating surfaces is achieved; *(vi) waiting*: the system is left to accommodate for 0.5 s; *(vii) back to the starting point*: the stage holding the glass counterface is moved back to its initial position; Importantly, with the contact kept open during steps *vi* and *vii*, the working solution can fully recover the frictional surface. For each test cycle, the static friction coefficient μ_s_ was computed by dividing the max tangential force *F*_s_ measured at the sliding inception point by the applied normal force *P*, as μ_s_ = *F*_s_/*P*; while the dynamic friction coefficient μ_d_ was computed as the average friction force *<F*_d_ *>* measured within the stabilized zone (middle of the sliding stock) divided by the applied normal force *P*, as μ_d_ = *< F*_d_ *>*/*P*. The impact of μHA on the friction coefficient was assessed by introducing different aliquots of microplates into the simulated synovial fluid, placed at the interface between the friction pair. Each experimental run involved 13 consecutive friction cycles: the initial 3 cycles served as a running-in phase without recording, while the subsequent 10 cycles were documented and saved for subsequent analysis to estimate the friction coefficients. After completing the 13th cycle, the 500 μL solution was removed using Kimwipes before initiating a new test run. To ensure reliability, each μHA configuration was tested three times, utilizing a new friction pair (Teflon and glass counterface) for each repetition.

### Degradation Studies of μHA

Degradation studies were performed by generating oxidative stress conditions. Briefly, about 1 000 000 μHA particles were separately resuspended in 1 mL of pure H_2_O_2_ and incubated at 37 ± 2 °C under constant rotation. At different scheduled time points (0, 1, 2, 4, 6, 24, and 48 h), μHA size and morphology were evaluated via the Multisizer 4 COULTER and an automated Leica DM5500 B research microscope (Leica Microsystems, Wetzlar, Germany). Using a similar approach, μHA degradation was studied by reproducing *in vitro* OA condition. Specifically, about 500 000 μHA were separately resuspended in 1 mL of simulated synovial fluid supplemented with H_2_O_2_ to reach a final concentration of 0.3 mM.^35^ The degradation state of μHA was evaluated by monitoring their size and morphology at specific time points (0, 5, 10, 30, 45 days) through the Multisizer 4 COULTER and automated Leica DM5500 B research microscope analysis.

### Biocompatibility and Anti-inflammatory Activity of μHA

μHA biocompatibility was assessed on two cell types that typically populate the joint capsule, namely, chondrocytes and fibroblasts. Specifically, ATDC5 cells, a murine chondrogenic cell line, were cultured at 37 °C in 5% CO_2_, in DMEM/F-12, GlutaMAX medium supplemented with 10% FBS and 1% penicillin/streptomycin. Human chondrocytes were cultured in poly-l-lysine-coated flask (2 μg/cm^2^, T-75) at 37 °C in 5% CO_2_ in Chondrocyte Growth Medium supplemented with 5% FBS, 1% penicillin/streptomycin, and 1% Chondrocyte Growth supplement. Fibroblast-like synoviocytes (FLS) were isolated from the paws of healthy mice following a l previously established protocol.^35^ FLS were cultured at 37 °C in 5% CO_2_, in DMEM high-glucose medium supplemented with 10% FBS and 1% penicillin/streptomycin. For the viability assay, cells at 80% confluence were seeded into 96-well plates at 5 × 10^3^ cells/well. After 24 h, cells were treated using different μHA/cell ratios (from 1:10 to 1:1). Cell viability was assessed via an MTT assay. Specifically, at the end of predetermined incubation times, 5 mg/mL of MTT solution in PBS buffer was added to each well, and the cells were incubated for 4 h at 37 °C. The formed formazan crystals were dissolved in ethanol, and absorbance was measured at 570 nm, using 650 nm as the reference wavelength (Tecan, Männedorf, Switzerland). The percentage of cell viability was assessed according to the following relation: Viability (%) = Abs_t_/Abs_c_ × 100, where Abs_t_ and Abs_c_ are the absorbance values of treated and untreated (control) cells, respectively.

The interaction between μHA and human chondrocytes was studied under confocal microscopy too. Briefly, 8000 human chondrocytes were seeded into a μ-Slide 8 Well high maintaining culturing conditions, as described above. These cells were treated with 8000 μHA, previously stained with WGA, for 24 h. After treatment, the culturing media were removed, and the cells were washed twice with PBS. Fixation was performed using a 3.7% solution of paraformaldehyde for 10 min; 3 washes with PBS were performed after cell fixation. Cells were stained for actin using Alexa Fluor 568 Phalloidin according to the vendor’s instructions . For all analyses, nuclei were stained using DAPI following the vendor’s instructions . A 63× objective was used, and a z-stack series was acquired with 19 steps of 1000 nm each. Images were captured using an A1-Nikon confocal microscope (Nikon Corporation, Japan). A maximum intensity projection image was generated by using NIS-Software (Nikon Corporation, Japan).

In addition, the anti-inflammatory activity of μHA was tested *in vitro*. Human chondrocytes were seeded in a poly-l-lysine-coated 24-well plate at a density of 7 × 10^4^ per well and left to grow until confluency. To mimic the OA environment, cells were stimulated with 0.3 mM H_2_O_2_ for 24 h and then treated with μHA (cell/particle ratio 1:1). After 24 h, the cell culture media were harvested, and the amounts of IL-6, IL-1β, and TNF-α were quantified by an ELISA kit following the manufacturer’s protocol. The optical density measurement of each well was conducted on a Tecan plate reader (Tecan Group AG, Männedorf, Switzerland) at 450 nm.

### Establishment of 3D Osteoarthritis Model with ATDC5 Cells

ATDC5 cells were cultured at 37 °C in 5% CO_2_, in DMEM/F-12, GlutaMAX medium supplemented with 10% FBS, and 1% penicillin/streptomycin. Spheroid formation was initiated by pelleting ATDC5, at a density of 125 000 cells per well, in 96-well round-bottom plates with Ultra Low Attachment surfaces. The cells were cultured in chondrogenic media (MEM-α, 5% FBS, ITS+ 1×, 10 ng/mL TGF-β3, 100 μM l-ascorbic acid, 100 nM dexamethasone) for 28 days. To ensure continuous chondrogenic differentiation, the medium was refreshed every 3 days throughout the entire culture period. To replicate the inflammatory environment of OA, 3D cartilage aggregates at day 28 were exposed to IL-1β (5 ng/mL) for 72 h in the presence or absence of μHA at different concentrations. Following the treatment, cell culture media were collected to evaluate the level of matrix degradation. The main components of the extracellular matrix, glycosaminoglycans (GAG) and collagen, are degraded under inflammatory conditions by ADAMTS (disintegrin and MMPs with thrombospondin motifs) and metalloproteinases (MMPs), respectively. Therefore, GAG released in the media was measured using the 1,9-dimethylmethylene blue (DMMB) assay. Media samples (100 μL) were mixed with 100 μL of DMMB working solution at room temperature. The absorbance was measured at 525 nm, and chondroitin sulfate was used as a standard. In addition, the activity of matrix metalloproteinases (MMPs) in the media was assessed using the fluorometric MMP-13 Activity Assay Kit.

### *In Vivo* Osteoarthritis Model via Repetitive Mechanical Loading

The PTOA model based on noninvasive repetitive joint mechanical loading, approved by the Vanderbilt Institutional Animal Care and Use Committee, was adapted from previous studies.^31^ Twenty-eight C57BL/6 mice were aged to 6 months and, following anesthesia with 3% isoflurane, were subjected to rigorous cyclic mechanical loading of 8.6 N per load, 250 cycles per session, each cycle lasting 2.5 s, with 3 loading sessions per week for 2 weeks using a TA ElectroForce 3100 (TA Instruments, New Castle, Delaware, USA). Cyclic loading was performed utilizing two form-fitting insets—one covering and stabilizing the kneecap and another holding the ankle in a flexed position of 135°. Specifically, the mold for the kneecap is a half-sphere cavity measuring 5 mm in diameter, and the mold for the ankle is a cavity shaped as an equilateral triangular, measuring 5 mm in diameter on each side. To clarify, our study does not employ an aging- or degeneration-driven model of primary osteoarthritis (OA). Instead, we use a noninvasive, load-induced model of OA, in which excessive joint loading serves as the initiating factor of pathology. This approach avoids surgical intervention and the confounding effects of permanent joint destabilization while remaining analogous to widely used models such as anterior cruciate ligament transection. Because joint overloading represents a defined mechanical insult, this model has been extensively validated by our group and others as a reproducible model of PTOA.^31,36–38^

All treatment groups were administered a single dose on the day after the first loading cycle. Specifically, mice received a single intra-articular injection of 10 μL of either saline, HYALGAN (10 mg/mL), or 10 kDa (10-P_25_) and 500 kDa (500-P_28_) μHA (both at 10 mg/mL).

### Inflammatory Gene Expression Analysis on PTOA Mice

The gene expression was analyzed from the synovial tissues of both knees per animal, with the primers and reagents listed in the Materials section. Under a surgical microscope, the knee synovium was dissected from the anterior, medial, and lateral compartments and homogenized using 5 mm TissueLyser stainless steel beads (Qiagen) and TRIzol (Thermo Fisher Scientific) in 2 mL tubes for 5 min at 30 Hz using the TissueLyser II (Qiagen). RNA was collected using the RNeasy mini-prep kit (Qiagen). The iScript cDNA RT kit (Bio-Rad) was used for cDNA production. Normalizing the mass of cDNA across samples, quantitative PCR was performed with 2× Universal SYBR Green Fast qPCR Mix, using RPL4 expression as a housekeeping gene. Primer pairs used for the detection of gene expression are listed in Table 1.

**Table 1:** 

Gene	Forward 5′ to 3′	Reverse 5′ to 3′
Rpl4	GCC AGG CCA GAA ATC ACA AA	TCC TTT CTT GCC TAC CGC TG
IL-1β	GCC ACC TTT TGA CAG TGA TGA G	GAC AGC CCA GGT CAA AGG TT
TNF-α	CCA CCA CGC TCT TCT GTC TA	GGC CAT TTG GGA ACT TCT CAT C
MMP-13	GGC CAG AAC TTC CCA ACC AT	GAG CCC AGA ATT TTC TCC CTC T

### Statistical Analysis

Data are displayed as mean ± standard error (*n* ≥ 3). Statistical tests employed either one-way ANOVA with a multiple comparisons test or two-way ANOVA, using GraphPad Prism 10. Differences were considered significant if *p* < 0.05.

## Results

### Fabrication of μHA

First, a photopolymerizable functional group was covalently introduced into the HA backbone, as depicted in Figure 1A, via hyaluronic acid (HA) methacrylation with glycidyl methacrylate (GM). HA chains with two distinct molecular weights, namely, 10 and 50 kDa, were functionalized by systematically changing both the HA:GM ratio (1:50 and 1:100) and reaction time (12 h, 2, and 5 days) to identify the most effective conditions. All methacrylate prepolymers obtained were characterized via ^1^H NMR, which confirmed the success of the reaction by revealing methacrylate peaks at 6.2, 5.8, and 1.9 ppm (Supplementary Figure 1B). The degree of methacrylation was determined by calculating the relative integrated intensities of methacrylate protons (peak at 1.9 ppm) and methyl protons in HA acetamide (peak at 2.1 ppm). As expected, the table in Supplementary Figure 1C illustrates that preserving the HA:GM ratio while extending the reaction time from 12 h to 5 days consistently resulted in an increase in the degree of methacrylation (DM) by almost twofold for both 10 and 50 kDa HA chains. Conversely, at fixed reaction times, increasing the HA:GM ratio resulted in a noticeable increase in DM, especially under the 5-day condition. To further investigate the effect of HA chain length and degree of methacrylation on the physicochemical properties of polymeric particles, we selected four polymer precursors as building blocks: 10 kDa HA-MA with 15% DM (10-P_15_); 10 kDa HA-MA with 25% DM (10-P_25_); 50 kDa HA-MA with 17% DM (10-P_17_); and 50 kDa HA-MA with 30% DM (10-P_30_).

**Figure 1:**
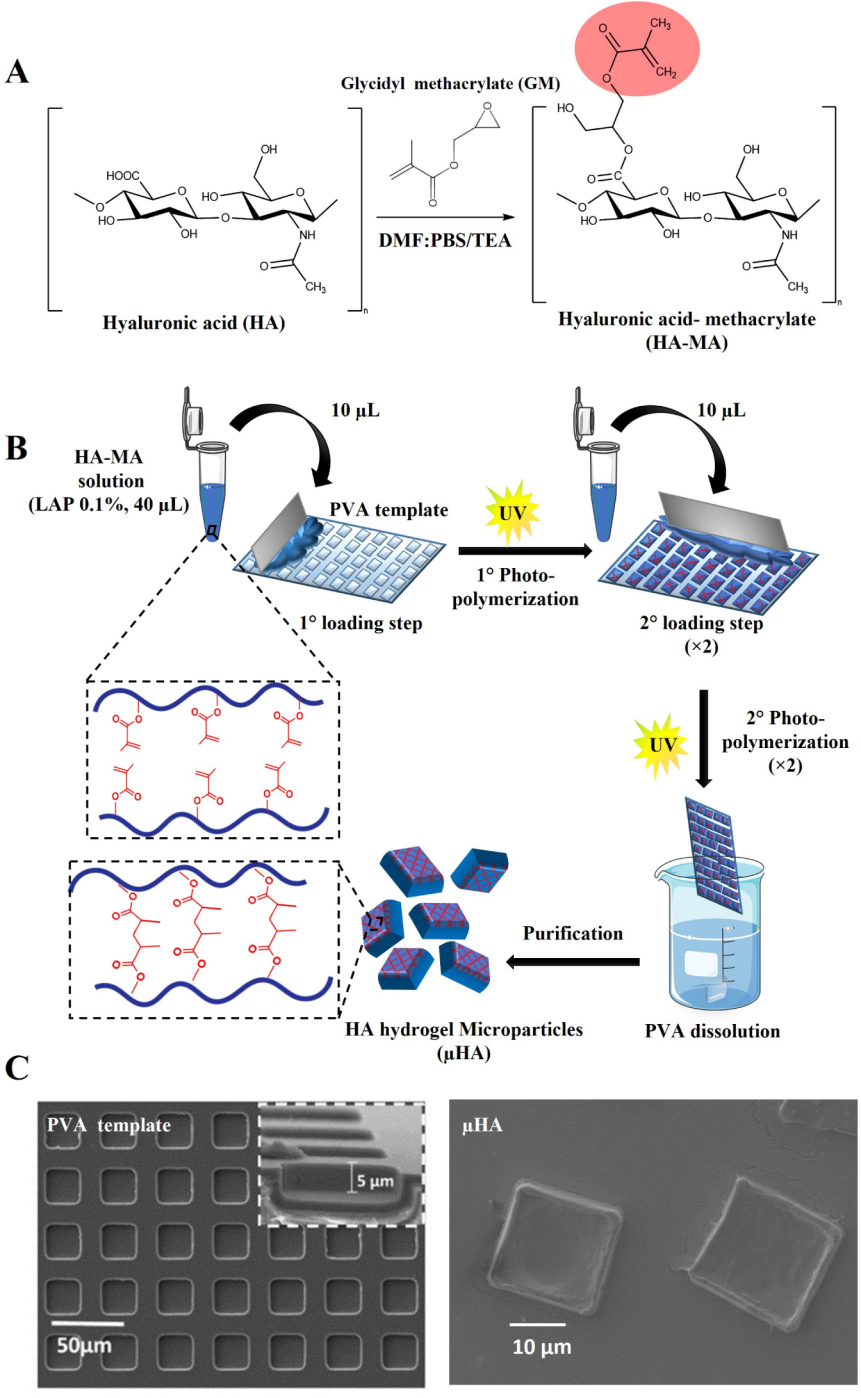
**Synthesis of HA-MA precursors and HA hydrogel microparticles (μHA).** **A.** Methacrylation reaction of hyaluronic acid (HA) with glycidyl methacrylate (GM) to generate photopolymerizable HA-MA precursors. **B**. Schematic representation of the fabrication process of μHA combining replica molding and multistep photopolymerization, in the presence of lithium phenyl-2,4,6-trimethylbenzoylphosphinate (LAP) as a photoinitiator. **C**. SEM analysis of a PVA sacrificial template, including a regular array of square wells with an edge length of 20 μm and a depth of 5 μm (***left***); individual μHA released upon dissolution of the PVA template (***right***) replicating the geometry of the original wells.

### Size Distribution and Fabrication Yield of μHA

A template-based approach was adopted to produce hydrogel microparticles with precise size and shape.^32^ Briefly, the four HA-MA precursors were mixed with the photoinitiator (LAP) in a water/glycerol (W/G) solution and spread over a PVA template to carefully fill a series of squared wells with an edge length of 20 μm and a depth of 5 μm (Figure 1B,C). Eventually, the PVA template was dissolved in water under constant stirring, and the HA-μHA microparticles were collected via centrifugation (Figure 1C, right).

To explore the impact of HA-MA concentration on the microplates, the fabrication process was carried out using HA-MA solutions at 5, 10, and 15% w/v (Supplementary Figure 2C and Figure 2). All resulting μHA were characterized using a Multisizer 4 COULTER, which counts the number of microparticles in solution and provides the corresponding size distribution (Figure 2B). The fabrication yield can also be estimated by calculating the ratio of the number of μHA produced per template to the total number of wells in a template. It is important here to note that, due to the nonspherical shape of the μHA, the Multisizer sizing appears as a distribution with a maximum corresponding to the average size of the particles’ characteristic dimensions. Figure 2B demonstrates that an increase in HA-MA concentration [5% (blue profile), 10% (red profile), and 15% (green profile) w/v)] and in HA-MA molecular weight [10 kDa (top row) to 50 kDa (bottom row)] resulted in narrower and higher peaks, suggesting an increase in fabrication yield and a decrease in particle dispersity. Generally, concentrations of 5% w/v HA-MA generated batches of μHA with a broad size distribution and low yields (blue profiles in Figure 2B). Additionally, the increase in both polymer concentration and molecular weight (MW) produced a distribution shifted toward a larger particle population, namely ∼8 μm for 10 kDa HA-MA and ∼10 μm for 50 kDa HA-MA. Indeed, since the actual geometry is dictated by the template, it is not surprising that the characteristic sizes of both 10 and 50 kDa HA-MA are nearly identical. The bar charts in Figure 2C summarize the yields for all 12 tested configurations. Significant differences appear only at low polymer concentrations of 5% w/v. Conversely, for concentrations of 10 and 15% w/v, the fabrication yield is around 60%, independent of the degree of polymerization and HA chain length.

**Figure 2:**
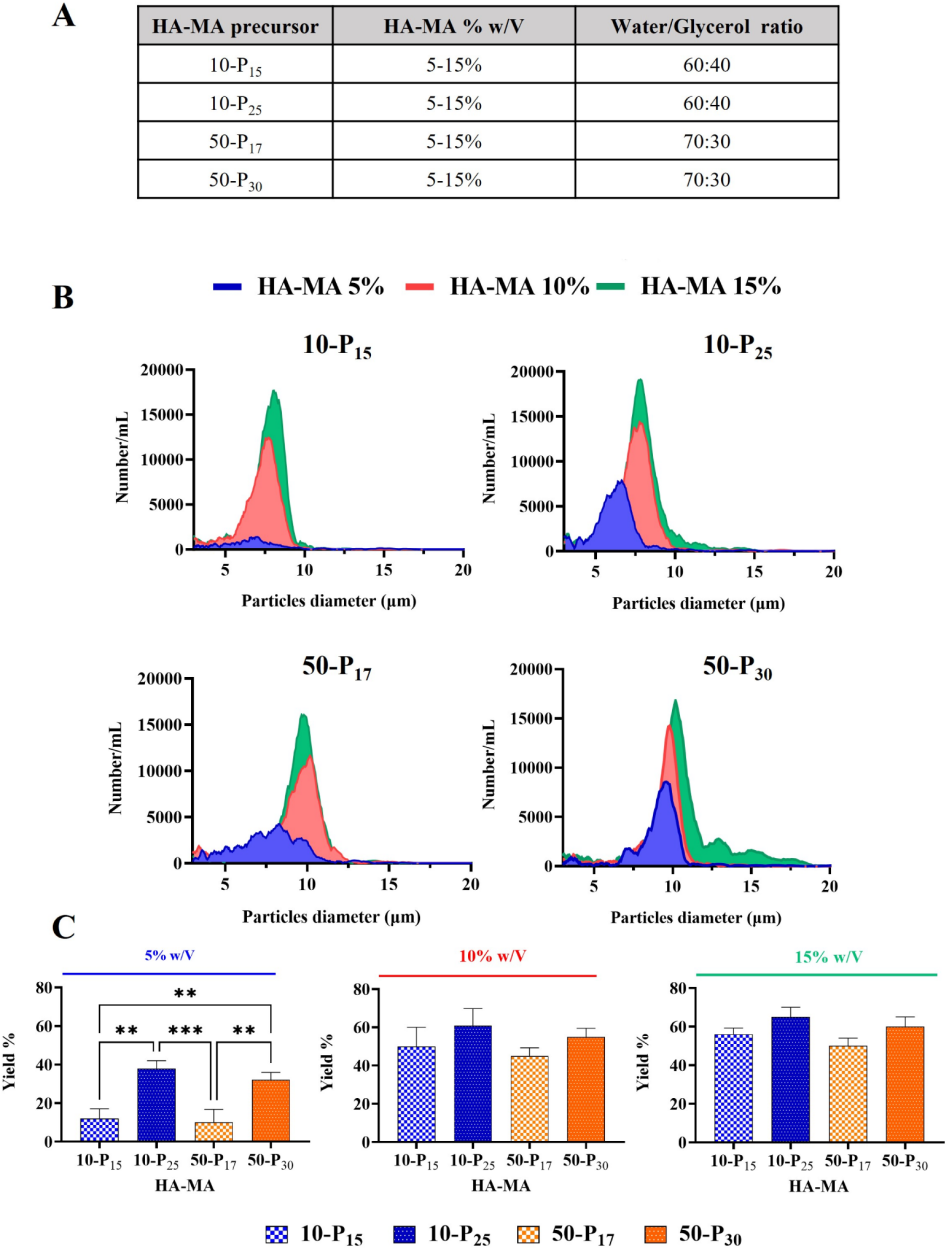
μHA size distribution and fabrication yield. **A.** HA-MA precursors used for the μHA fabrication, with varying molecular weights (MW: 10 and 50 kDa) and degrees of methacrylation (DM). HA-MA precursors were dissolved in a water–glycerol (W/G) solution containing 0.1% (w/v) of the photoinitiator agent LAP. **B**. Size distribution of μHA fabricated with four different HA-MA precursors at three different concentrations (5%: blue, 10%: red, 15%: green w/v). **C.** Yield of μHA samples fabricated as a function of the HA-MA precursors and concentrations.

Additionally, SEM analyses were performed on samples dried overnight at room temperature or samples dehydrated with ethanol. Microparticles fabricated using the highest amount of prepolymer (15% w/v) were selected for this analysis. In the SEM pictures, dried μHA (10-P_25_ and 50-P_30_ as building blocks) showed a well-defined square shape, with an edge length of 20 μm, but without a discernible height (Supplementary Figure 4A). These particles, as soft hydrogels, retain a large amount of water, with respect to the mass of the polymer. Thus, when they lose water, they collapse and appear “flat”. Conversely, following an ethanol dehydration process where the water entrapped inside the microparticles is gradually displaced, the collapse of the gel is prevented, and the microparticle height can be readily appreciated (Supplementary Figure 4B). However, the use of ethanol causes significant bulk shrinkage of the μHA, approximately 50%, resulting in an edge length of 12 μm and a height of 3 μm.

### Mechanical Characterization of μHA

Nanoindentation experiments were conducted to assess the apparent Young’s modulus under compression of μHA, employing a commercially available nanoindenter (Figure 3A). All of the mechanical characterizations were conducted on microparticles derived from the two HA-MA precursors with the highest degrees of polymerization, 10-P_25_ and 50-P_30_. These μHA were associated with the highest fabrication yield and the most accurate reproduction of the original geometry. Figure 3B provides representative load-indentation curves for the two considered μHA configurations, illustrating a loading phase up to a maximum penetration depth of ∼800 nm, followed by a holding phase at around 0.3–0.4 μN, and an unloading phase reaching back to 0 μN. At least 10 load-indentation curves were generated for each μHA configuration. These curves were then fitted with the Hertz’s equation to extract the Young’s modulus of the indented μHA, considering only the loading phase up to a penetration depth of 400 nm (dashed green line, Figure 3B), in order to minimize any possible effects associated with the substrate. Figure 3C shows that in the microhydrogel network, an increase in HA molecular weight is responsible for an increase in particle stiffness, with resulting Young’s moduli comparable to those obtained for the corresponding macrogels (29.3 ± 8.8 kPa vs ∼59 kPa for 10-P_25_; 168.5 ± 73.5 kPa vs ∼125 kPa for 50-P_30_), as shown in Supplementary Figure 3D. In addition to static characterization, dynamic tests were conducted to assess the viscoelastic properties and potential mechanical damping behavior of μHA. A sinusoidal force was applied to the microplates at increasing frequencies (1, 2, 4, 10 Hz). The phase difference between input (force) and output (deformation) was recorded over time to extract the loss parameter tan δ, representing the energy dissipation in a material under cyclic load. Figure 3D shows the progressive increase in the loss parameter from ∼0.03 to ∼0.15 with the frequency of the applied load for both HA-MA precursors. At 1 Hz, tan δ values ranged between 0.03 and 0.06.

**Figure 3:**
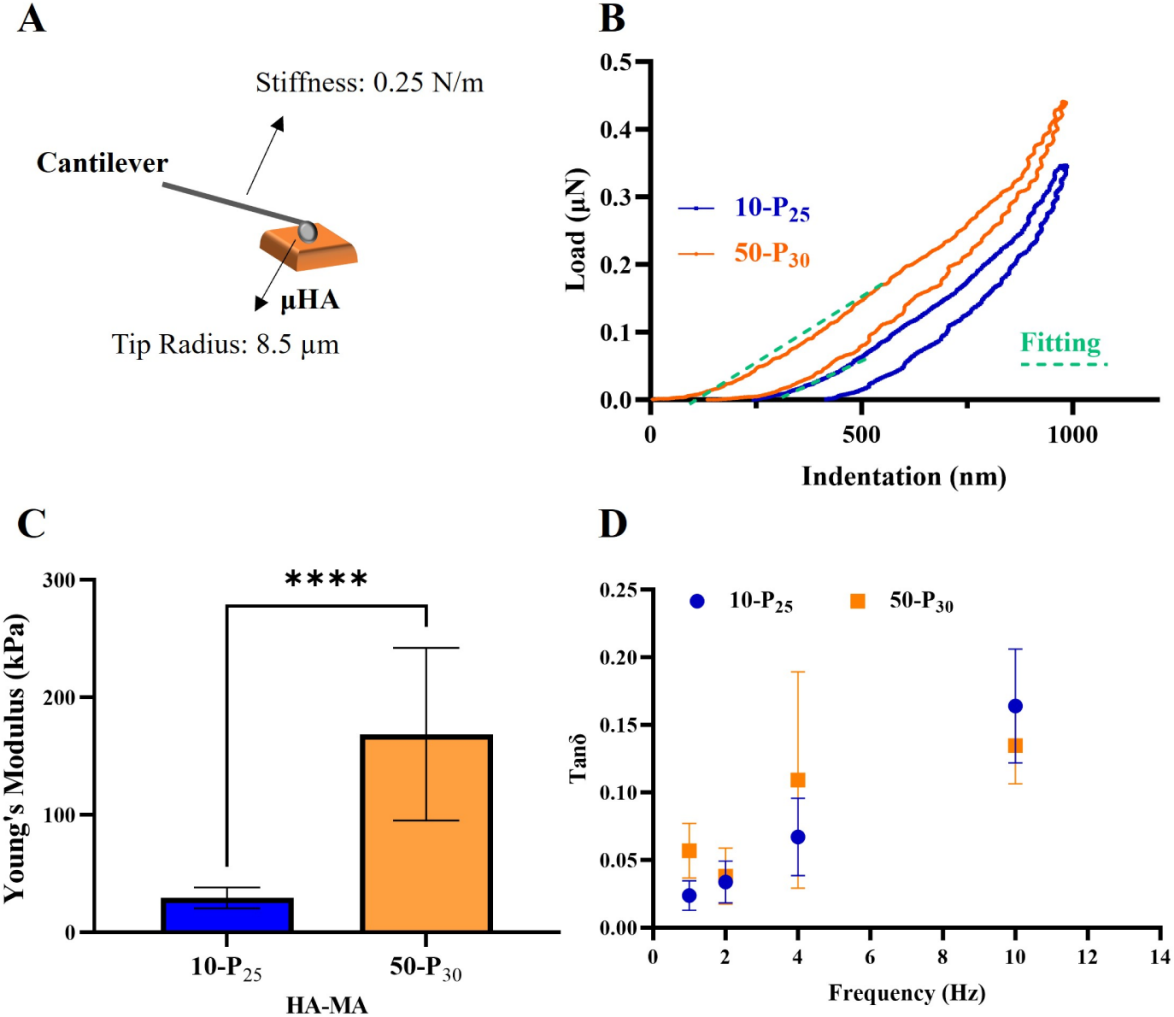
μHA mechanical characterization. **A.** Schematic representation of a nanoindenter’s tip equipped with an 8.5 μm spherical probe and a 0.250 N/m stiff cantilever in contact with the surface of a μHA. **B.** A representative load–indentation curve for a single μHA. The apparent Young’s modulus was estimated by fitting the loading portion of the indentation curves using the conventional Hertz theory (green dashed line). **C.** Apparent Young’s modulus for two different μHA configurations (10-P_25_ and 50-P_30_). **D.** Mechanical damping of μHA upon cyclic loading as a function of the frequency. Statistical analysis via one-way ANOVA: * indicates *p* < 0.05, ** indicates *p* < 0.01, *** indicates *p* < 0.001, and **** indicates *p* < 0.0001. “No significance” is not indicated on the graphs.

#### Tribological Characterization of μHA

Then, the tribological properties of μHA were assessed using a custom-built two-axis tribometer.^33^ Supplementary Figure 5A provides a schematic representation of the device, comprising two main units—one for system driving and operation and the other for measuring relevant forces. The experimental procedure involved following 8 specific steps, as depicted in Supplementary Figure 5B and detailed in the Materials and Methods section. In step 3, characteristic friction coefficient curves were generated under different experimental conditions by measuring the tangential forces where the counter glass surface is pushed against the Teflon rod with a normal load *P* = 5.8 N and slid over a 20 mm distance at a speed of 1 mm/s (Figure 4A,B left and Supplementary Figure 5C). Specifically, the static friction coefficient μ_s_ (= *F*_s_/*P*) is linked to the inception point, while the dynamic friction coefficient μ_d_ (= <*F*_d_>/*P*) is associated with the “stabilized zone”. Figure 4A,B (***left***) shows the typical friction coefficient curves for the two tested μHA configurations (10-P_25_ and 50-P_30_) at four different concentrations, namely 0, 0.6 × 10^5^, 1.2 × 10^5^, and 6 × 10^5^ μHA/mL. The native simulated synovial fluid with no particles was identified as an SF solution with 0 μHA/mL. The friction curves present similar trends, clearly documenting a peak (static friction coefficient μ_*s*_) followed by a stable phase (dynamic friction coefficient μ_d_). As shown by the bars in the chart of Figure 4A,B, both the static and dynamic coefficients of friction were observed to decrease by approximately 20% in the presence of μHA independent of particle concentration.

**Figure 4:**
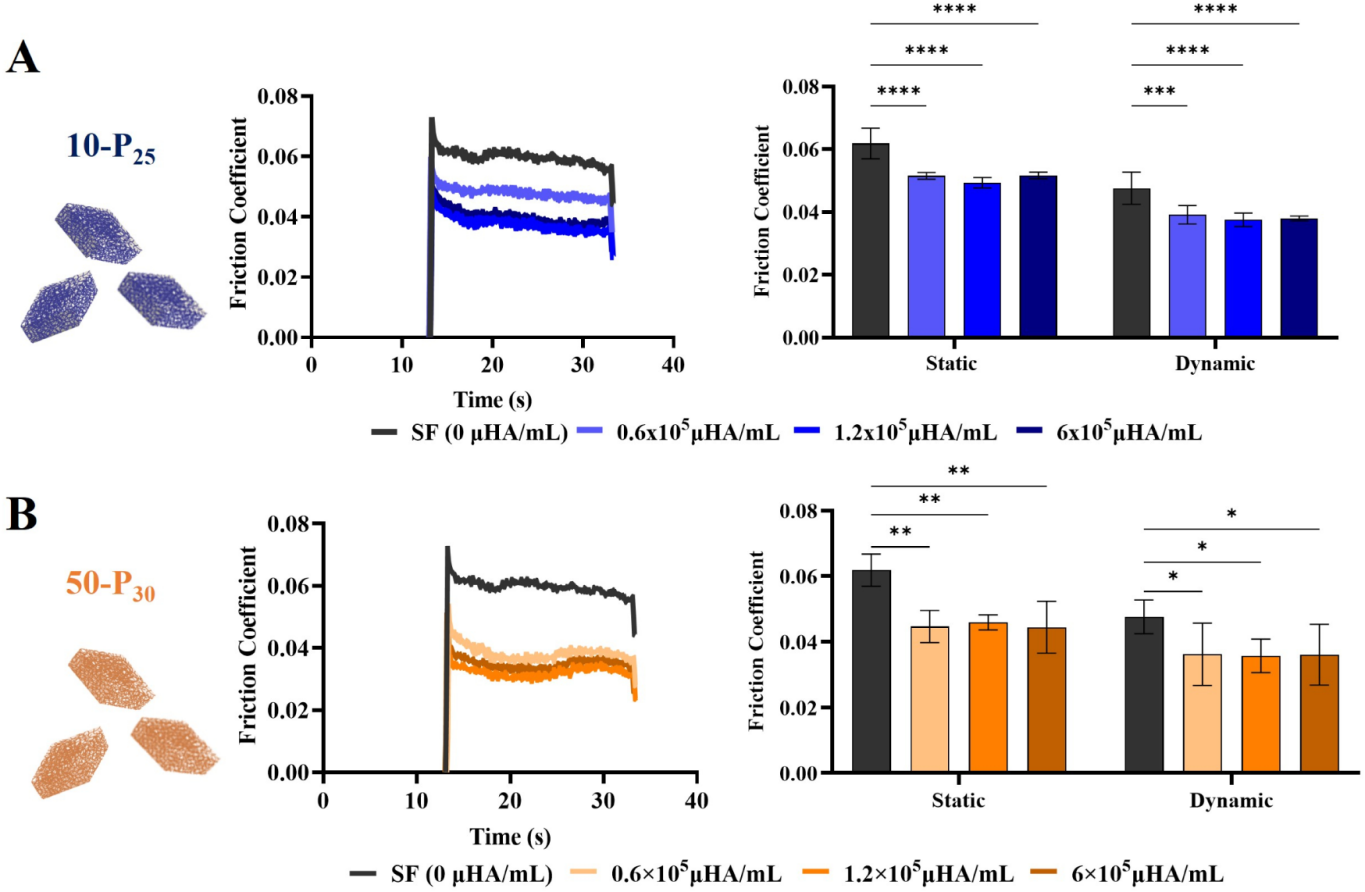
μHA tribological characterization. **A.** Representative curves for the coefficients of friction of 10-P_25_ μHA dispersed in simulated synovial fluid at different concentrations (***left***) and corresponding static and dynamic friction coefficients (***right***). **B.** Representative curves for the friction coefficients of 50-P_30_ μHA dispersed in simulated synovial fluid at different concentrations (***left***) and corresponding static and dynamic friction coefficients (***right***). Results are presented as mean ± SD (*n* = 5). Statistical analysis via two-way ANOVA: * indicates *p* < 0.05, ** indicates *p* < 0.01, *** indicates *p* < 0.001, and **** indicates *p* < 0.0001. “No significance” is not indicated on the graphs.

#### Degradation Studies of μHA

Considering the rapid degradation of free HA chains in osteoarthritic joints triggered by reactive oxygen species generated by inflamed chondrocytes, we assessed the degradation of μHA under oxidative conditions.^19^ To this end, the microparticles were incubated in simulated synovial fluid (SF) enriched with 0.3 mM H_2_O_2_ at 37 °C. Degradation was assessed by monitoring, at predetermined time points, the particle number, size, and morphology via Multisizer Coulter Counter and microscopy analyses. As illustrated in Figure 5A,B, μHA derived from both HA-MA precursors (10-P_25_ and 50-P_30_) exhibited excellent stability under oxidative conditions. Over the course of 45 days, the size distribution profile generated by the Multisizer Coulter Counter underwent modest variations (Figure 5A). As more clearly quantified in Figure 5B, the number (green curve) and size (black curve) of μHA in solution did not change in a statistically significant manner over the entire observation period.

**Figure 5:**
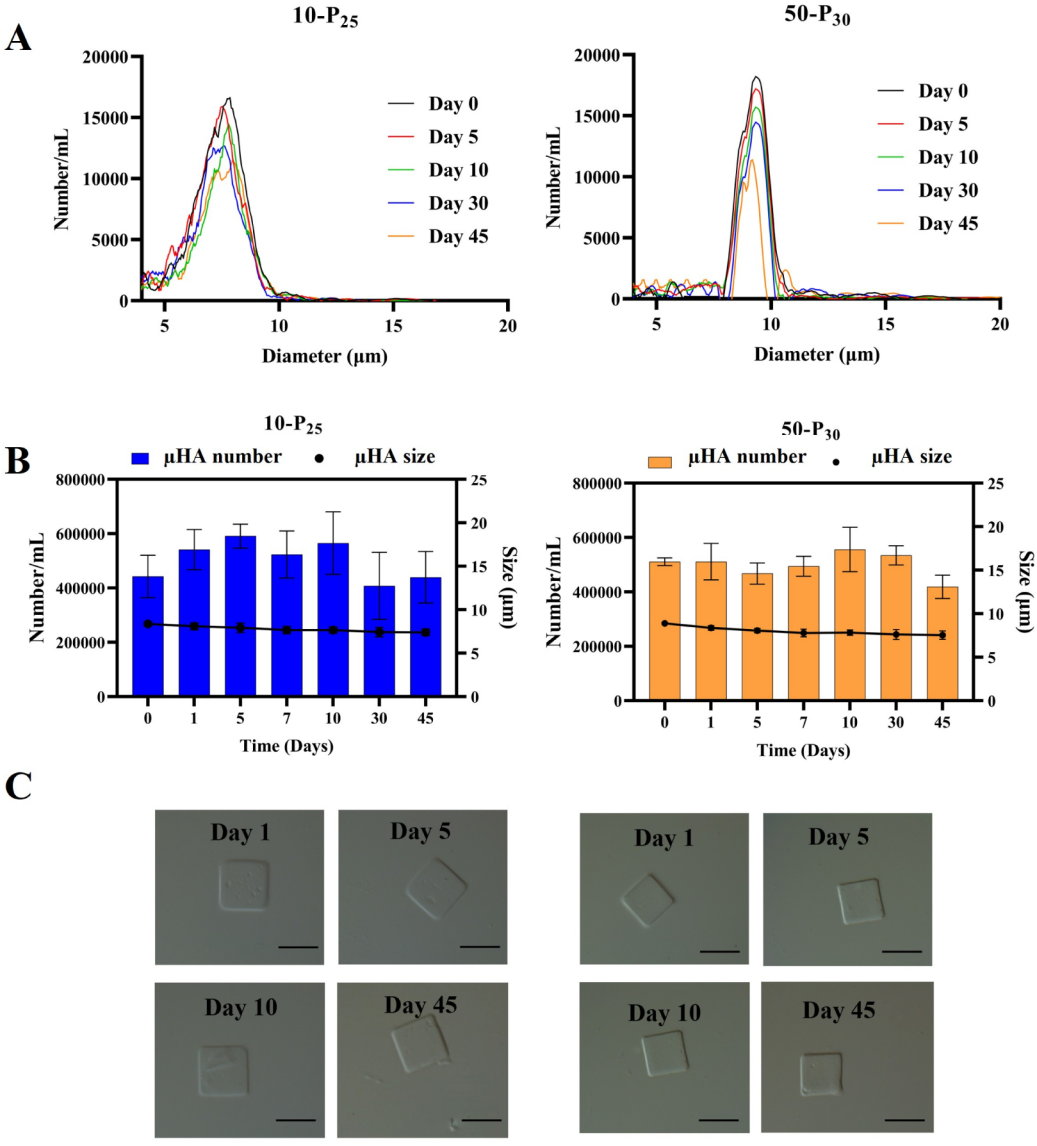
μHA degradation under oxidative stress conditions. **A.** Size distribution analysis via a Multisizer for μHA at predetermined time points. **B**. Number (number/mL) and average size of μHA, determined via Multisizer at different time points. **C.** Microscopy analysis of μHA at predetermined time points. For all of the experiments, 10-P_25_ and 50-P_30_ μHA were incubated with 0.3 mM H_2_O_2_ in simulated synovial fluid (SF). (Scale bar: 20 μm).

The degradation profile of μHA was also assessed under extreme oxidative stress conditions, corresponding to microparticle incubation in pure H_2_O_2_. The 10-P_25_ μHA were found to be stable for approximately 4 h, as shown in Supplementary Figure 6A. After this time point, the size and number of μHA in solution, as measured via the Multisizer Coulter Counter, were observed to rapidly decrease. These observations are also supported by bright-field microscope images documenting a well-defined 20 μm square shape of the μHA up to 4 h, followed by a sudden collapse and shrinkage of the particle (Supplementary Figure 6A, right). An increase in the molecular weight of the precursor, from 10-P_25_ HA-MA to 50-P_30_ HA-MA, resulted in slightly longer particle stability. As shown in Supplementary Figure 6B, the 50-P_30_ μHA resisted oxidative degradation for up to 6 h, with no significant changes in particle number and size.

#### Biocompatibility and *In Vitro* Anti-inflammatory Activity for μHA

The 10-P_25_ μHA were selected for the following characterizations and experiments because of their lower viscosity, which facilitates the spreading of the paste on the PVA template, lower mechanical stiffness, and more favorable degradation profile. Thus, the biocompatibility of 10-P_25_ μHA was assessed *in vitro* using ATDC5 cells, FLS, and human chondrocytes. Cells were exposed to various particle-to-cell ratios for 24, 48, and 72 h. Cytotoxicity was quantified via an MTT assay. Cell viability was unaffected by the presence of the particles up to a 1:1 ratio and 72 h (Figure 6B for human chondrocytes and Supplementary Figure 7A,B for ATDC5 and FLS). Note, as shown in the fluorescence microscopy image (Figure 6A), that the 20 μm μHA is comparable in size to chondrocytes and is not expected to be internalized.

**Figure 6:**
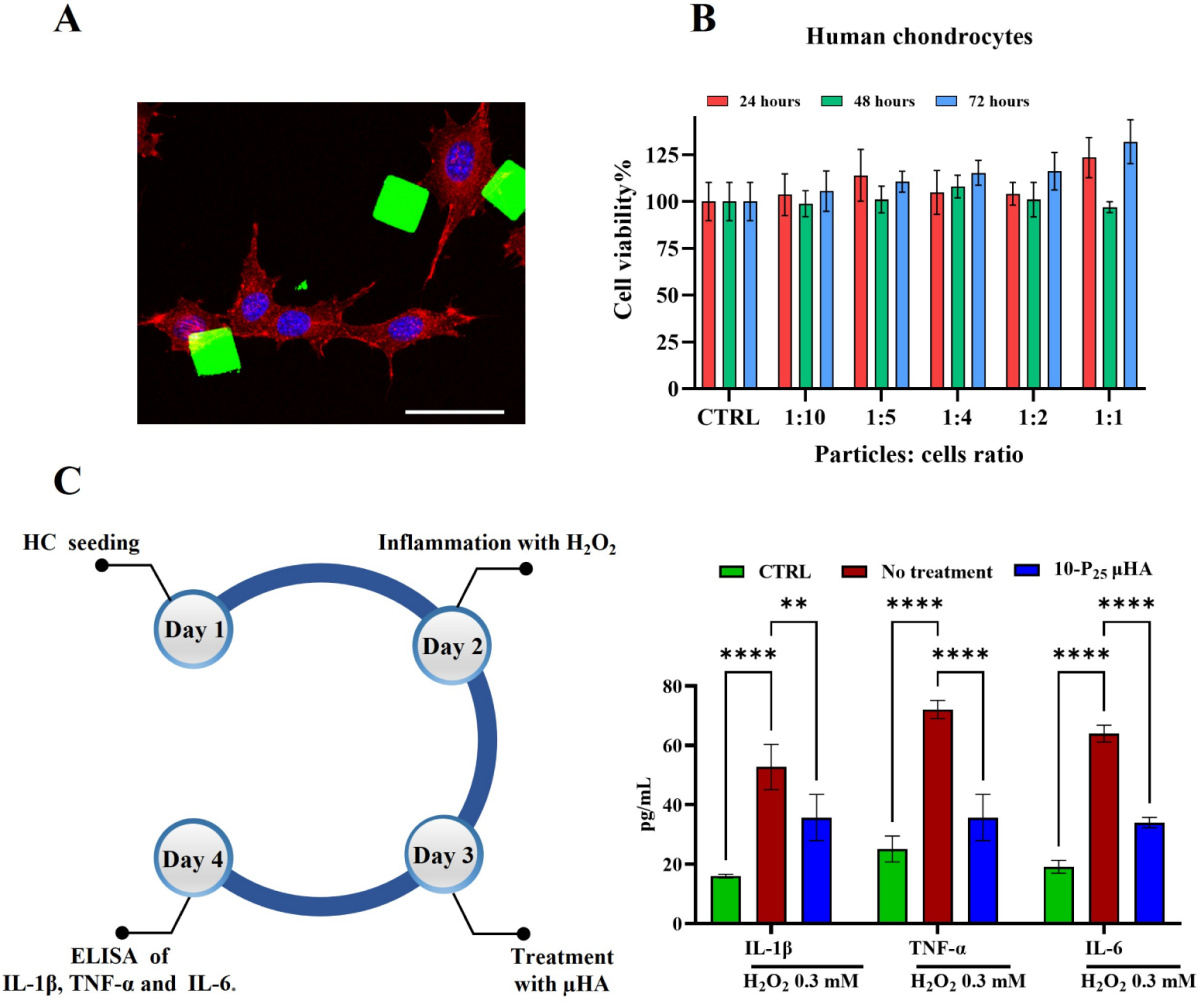
μHA *in vitro* biocompatibility and anti-inflammatory activity. **A**. Confocal imaging (in maximum intensity projections) of a culture of human chondrocytes treated with μHA fluorescently stained on *N*-acetyl-d-glucosamine (red signal: actin; green signal: μHA; blue signal: nuclei. Scale bar: 50 μm). **B.** The viability of human chondrocytes upon incubation with different amounts of 10-P_25_ μHA for 24, 48, and 72 h. **C.** On the left, experimental setup for the evaluation of μHA anti-inflammatory activity using human chondrocytes inflamed with H_2_O_2_ to mimic the OA environment. On the right, effect of H_2_O_2_ exposure on IL-6, TNF-α, and IL-1β secretion from human chondrocytes in the presence or absence of μHA. Statistical analysis via two-way ANOVA: * indicates *p* < 0.05, ** indicates *p* < 0.01, *** indicates *p* < 0.001, and **** indicates *p* < 0.0001. “No significance” is not indicated on the graphs.

To investigate the μHA anti-inflammatory activity, human chondrocytes were stimulated with 0.3 mM H_2_O_2_ for 24 h, mimicking OA ROS conditions. Then, μHA were incubated with the stimulated cells at a 1:1 cell-to-particle ratio for an additional 24 h. At the end of the treatment, the cell culture media were harvested, and the amounts of IL-6, IL-1β, and TNF-α were quantified by ELISA. As depicted in Figure 6C, exposure to H_2_O_2_ led to a significant increase in all cytokine levels for the untreated group compared to the unstimulated cells (CTRL). Specifically, IL-6 increased from 19.2 ± 2.2 to 64 ± 2.8 pg/mL; TNF-α from 25.1 ± 4.4 to 72.1 ± 3.1 pg/mL; and IL-1β from 16.0 ± 0.6 to 52.76 ± 7.638 pg/mL. The addition of μHA significantly reduced cytokine release, with IL-6 levels at 34.1 ± 1.7 pg/mL, TNF-α at 34.3 ± 7.2 pg/mL, and IL-1β at 20.4 ± 3.5 pg/mL (see Supplementary Figure 8 for statistical significance).

Moreover, ATDC5-derived chondrocyte spheroids, which form 3D cartilage aggregates, were used to confirm the chondroprotective properties of μHA (Figure 7A). These spheroids were grown for 1 month to ensure that the extracellular matrix was fully formed. To replicate the inflammatory environment associated with OA, ATDC5-based 3D cartilage aggregates on day 28 were exposed to IL-1β (5 ng/mL) for 72 h, with or without 10-P_25_. At the conclusion of the treatment, cell media were collected to assess the extent of matrix degradation. In inflammatory conditions, the primary extracellular matrix components, GAG and collagen fibers, are degraded by ADAMTS and matrix metalloproteinases (MMP), respectively. Specifically, the analyses included measuring GAG content in the media to assess GAG release/degradation and determining the activity of MMP-13, which is known to mediate type II collagen breakdown. As shown in Figure 7B, IL-1β exposure significantly increased the level of GAG release and MMP-13 activity in the media, indicating matrix degradation. Media analysis further demonstrated that both HA formulations provided protective effects against IL-1β-induced matrix degradation. Notably, the presence of μHA reduced GAG release by approximately 30%, though there was not a notable dose response.

**Figure 7:**
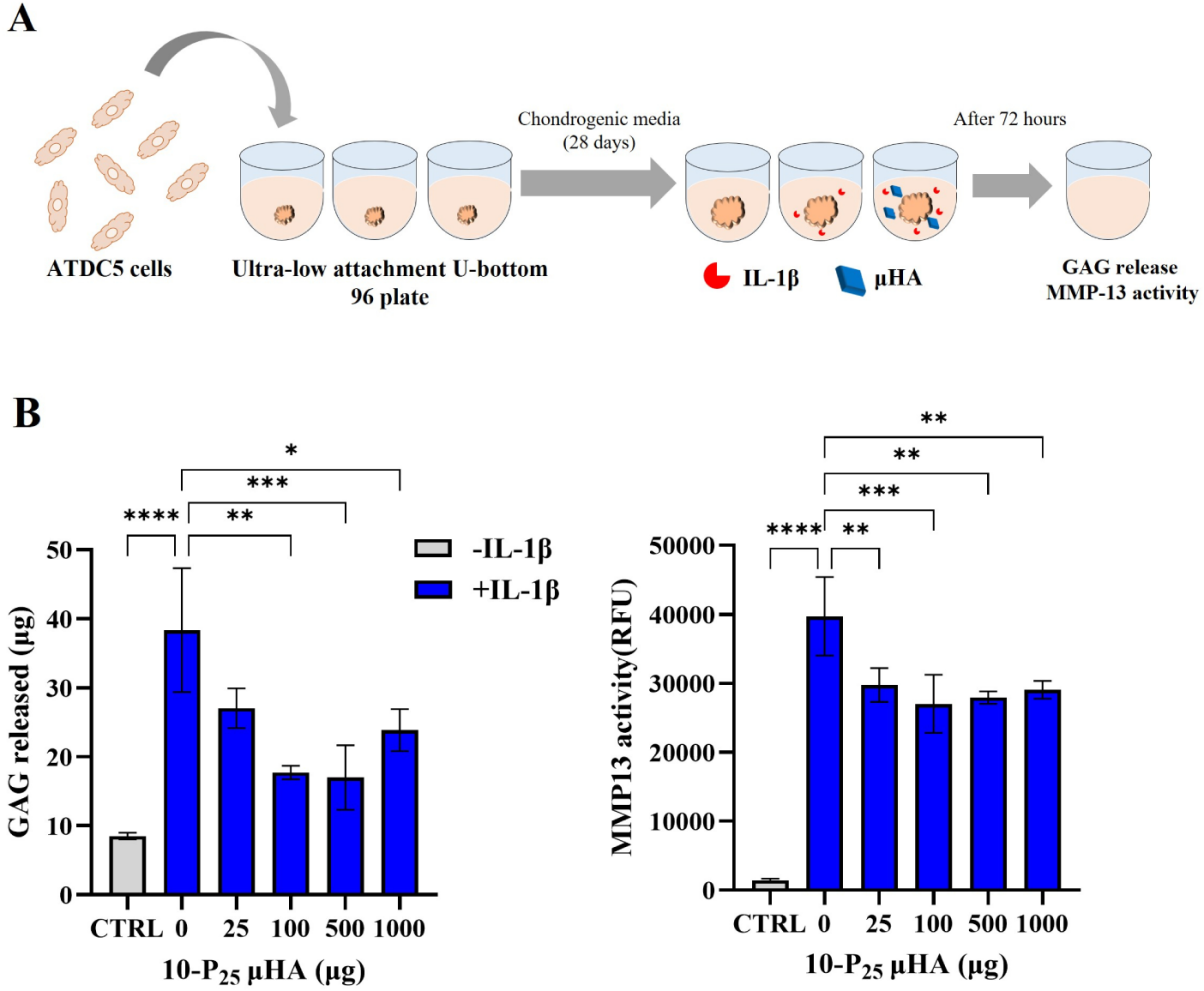
μHA chondroprotective effect on ATDC5 aggregates prestimulated with IL-1β. **A.** Schematic representation of the experimental procedure to develop an *in vitro* three-dimensional model of OA to assess the chondroprotective effect of μHA. **B**. GAG release and MMP-13 activity in the media of untreated ATDC5 aggregates, prestimulated with IL-1β and exposed to different amounts of 10-P_25_ μHA (10 kDa) for 72 h. Results are presented as mean ± SD (*n* = 3). Statistical analysis was performed via one-way ANOVA: * indicates *p* < 0.05, ** indicates *p* < 0.01, *** indicates *p* < 0.001, and **** indicates *p* < 0.0001. “No significance” is not indicated on the graphs.

#### Therapeutic Activity in a Murine Model of Early-Stage PTOA of μHA

Next, a 2-week *in vivo* study was conducted using a rigorous murine model of early-stage PTOA. Specifically, 6-month-old C57BL/6 mice were subjected to a knee joint cyclic mechanical loading protocol of 8.6 N, 250 cycles, for 3 times per week to induce PTOA. Following the first cycle of mechanical loading, a single intra-articular dose of HA (10 mg/mL, 10 μL) was administered into each knee of the mice in the form of 10-P_25_ μHA, the commercial product HYALGAN, which has a molecular weight of approximately 500 kDa, and the 500-P_28_ μHA, for comparison. The morphological, biocompatibility, and chondroprotective properties of the 500-P_28_ μHA are provided in the Supporting Information. After 2 weeks of mechanical loading (early PTOA model), qPCR was employed to assess the expression of genes associated with PTOA progression in the synovial tissue (Figure 8A), including specifically the proinflammatory cytokines IL-1β, TNF-α, and MMP-13. The data presented in Figure 8B, supported by the computed *p*-values listed in Supplementary Figure 9, show that μHA (blue triangles and green circles) significantly reduced IL-1β and TNF-α expression compared to knees treated with saline (red hexagons) and HYALGAN (red rhombuses). Additionally, no statistically significant difference was found in the expression of both cytokines between untreated mice and mice treated with the two μHA formulations. The expression of MMP-13, which is a downstream inflammatory mediator, was upregulated in the saline group, but no treatment was able to produce a significant reduction in its expression.

**Figure 8:**
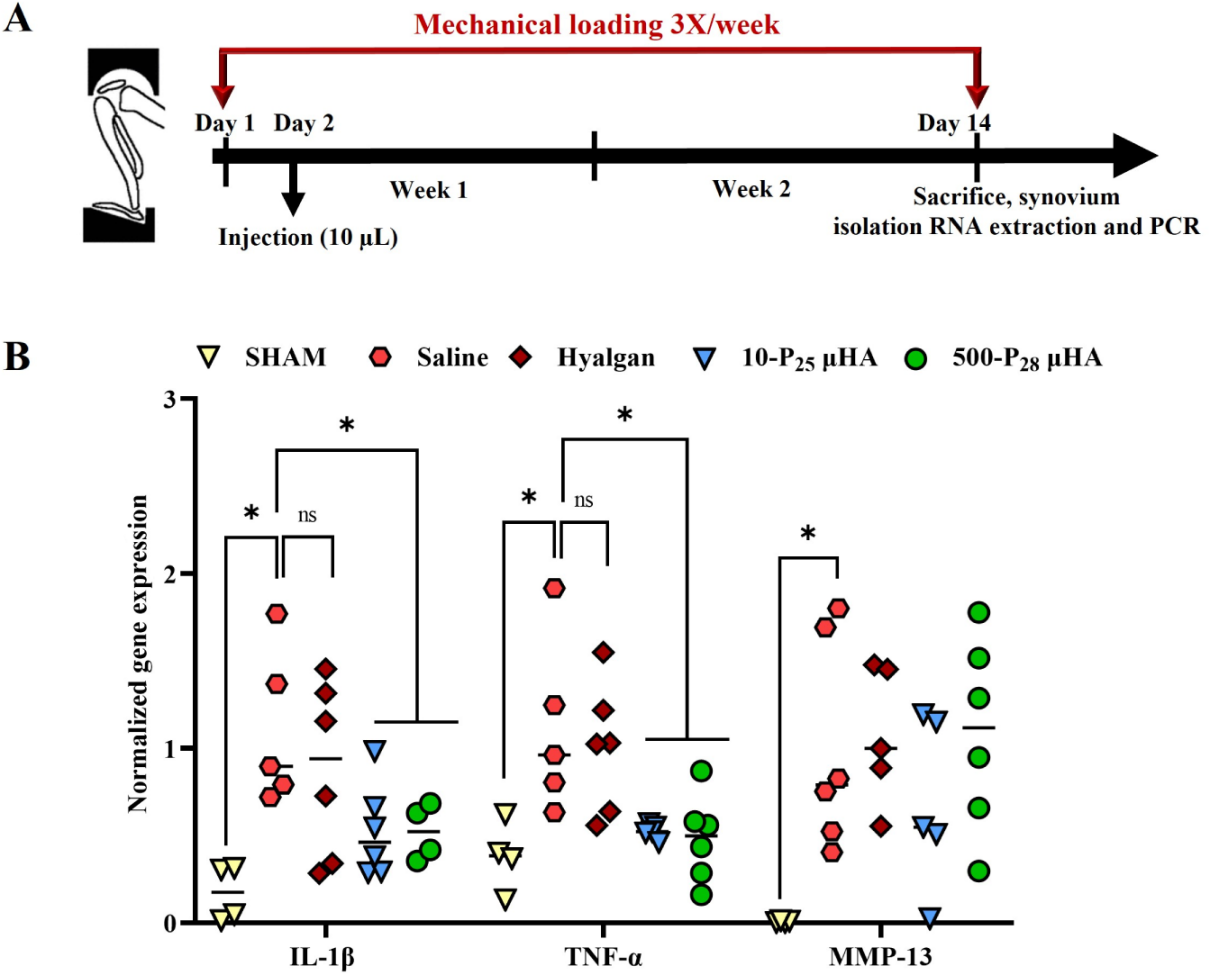
**A.** Proinflammatory gene expression in a PTOA mouse model. **A.** Schematic of the loading fixture used in the mechanical loading of mouse knee joints to induce PTOA (3 loading sessions per week for 2 weeks using a TA ElectroForce). All treatment groups were administered once, on the day after the first loading cycle. Specifically, mice received a single intra-articular injection of 10 μL of saline, HYALGAN (10 mg/mL), or 10-P_25_ and 500-P_28_ μHA (both at 10 mg/mL). **B**. *In vivo* expression of IL-1β, TNF-α, IL-6, and MMP-13 measured by qPCR (for each treatment group *n* = 6, while for the healthy group *n* = 4). Statistical analysis via one-way ANOVA, corrected for multiple comparisons by controlling the false discovery rate with a two-stage, step-up Benjamini–Krieger–Yekutieli method: *p < 0.05 and **p < 0.01, while “no significance” is indicated as “ns”.

## Discussion

Building on the fundamental role of endogenous hyaluronic acid in joints, we developed HA-based microscopic gels (μHA) with dual functionality: lubrication enhancers and antioxidant/anti-inflammatory agents. In the present work, four different HA-MA prepolymers were engineered by covalently introducing methacrylate groups into the backbone of low- (10 kDa) and high- (50 kDa) molecular-weight HA chains, with a low and high degree of methacrylation. Consistent with previous studies, we confirmed that increasing the stoichiometric amount of methacrylate precursors and extending the reaction time led to a higher degree of methacrylation.^39^ Then, a template-based microfabrication approach was employed to realize μHA. This required the formation of an aqueous solution with the HA-MA prepolymer of choice and carefully spreading it to fill an array of microscopic wells carved into a sacrificial PVA template. This method yielded μHA with a square base of 20 μm and a height of 5 μm. Notably, the μHA geometry was uniquely defined by the geometry of the template, which can be readily modified.^32^ It is important to note that the only intra-articular particle-based formulation approved for OA treatment is Zilretta, a PLGA-based microsphere formulation of triamcinolone acetonide. These microparticles are spherical, 35–55 μm in diameter, and engineered for extended corticosteroid release, with a smooth surface and uniform geometry to ensure injection stability and reduce joint irritation. The 75:25 PLGA copolymer composition allows for controlled degradation and sustained drug release, leading to reduced systemic exposure, which is particularly advantageous for corticosteroid-sensitive populations, such as individuals with diabetes. In contrast, the μHA particles proposed in this study differ fundamentally from Zilretta in terms of material composition, geometry, and intended mechanism of action. μHA are composed of hyaluronic acid, are not drug loaded in their current formulation, and exhibit a distinctive microplate (5 μm thick) geometry rather than a spherical one. This geometry provides multiple benefits over rigid PLGA microspheres. μHA plates can deform under the joint load, which helps reduce friction. Their flexible shape also allows them to serve as mechanical barriers at compressed cartilage interfaces. Additionally, their small, flat structure makes them easily injectable and capable of dispersing more uniformly within synovial fluid. The high surface-area-to-volume ratio and anisotropic surface of μHA further promote adhesion to inflamed or damaged joint tissues, particularly in areas with abundant fibrin, collagen, or exposed extracellular matrix components. These features collectively suggest that μHA may offer unique mechanical and biological advantages over traditional spherical particles in OA therapy.

In addition to precisely controlling particle size and shape, we demonstrated that the mechanical properties of μHA could also be finely tuned by modulating the concentration, molecular weight , and degree of methacrylation of the HA-MA prepolymers. The mechanical properties of μHA were consistent with previously observed trends for HA-based macroscopic hydrogel systems.^40^ Specifically, the elastic modulus increased with both the molecular weight and the degree of methacrylation. For example, the Young’s modulus of the 50 kDa μHA with a high degree of methacrylation (50-P_30_) was ∼170 kPa, significantly much higher than that of the 10 kDa μHA with a low degree of methacrylation (10-P_25_), which was ∼30 kPa. Also, dynamic tests assessing the viscoelastic properties of μHA revealed frequency-dependent damping behavior, with the tan δ values increasing with the load frequency. This behavior may be beneficial for dynamic applications, such as within the two mating surfaces of a joint, where the material must effectively respond to cyclic loading.^41^ Tribological studies, performed with a pin-on-plate system, showed that incorporating μHA into synovial fluid significantly reduced both static and dynamic friction coefficients by approximately 20%. This suggests that μHA enhances the lubricating properties of the synovial fluid placed between a friction pair. Notably, the tribological performance was largely independent of μHA concentration, indicating that even at low particle densities, μHA effectively improves lubrication. Moreover, the hydrogel nature of μHA allows them to absorb synovial fluid, enhancing their load-bearing capacity and ability to reduce friction.^42^

While a 20% decrease in the coefficient of friction may appear modest in absolute terms, it is consistent with reductions reported in recent studies employing a variety of approaches. For instance, Lei et al. demonstrated that 200 μm lipomicrospheres, obtained by assembling 100 nm liposomes with 74 kDa hyaluronic acid chains, reduced the coefficient of friction from 0.06 to 0.04 via a rolling lubrication mechanism.^43^ Similarly, Han et al. reported approximately a 30% reduction from 0.027 to 0.019 using photo-cross-linked methacrylate gelatin hydrogel microspheres with a quasi-spherical shape and a mean size of approximately 150 μm.^44^ Notably, the proposed μHA reduces the coefficient of friction from 0.05 to 0.038. It is also important to note that our tribological characterizations were performed by sliding a simulated synovial fluid enriched with μHA between two rigid solid surfaces. This is a configuration mimicking extreme joint conditions and is known to increase friction. Indeed, rigid surfaces cannot sustain interstitial fluid pressurization, leading to increased surface contact and, consequently, higher friction. Under such conditions, the moderate variation in friction is primarily due to the lack of complementary deformation and lubrication mechanisms that are intrinsic to cartilage-on-cartilage articulation. In contrast, native articular cartilage is porous, and this porosity plays a critical role in modulating friction by enabling interstitial fluid flow at the articulating interface, as elegantly demonstrated by Shi et al.^45^ Finally, it is worth emphasizing that wear and tissue damage are cumulative processes that develop over millions of joint loading cycles. Even a seemingly modest 20% reduction in the coefficient of friction, if maintained over time, could confer significant long-term benefits. However, these considerations remain speculative and should be validated in future studies, ideally involving larger animal models where gait analysis could elucidate the sustained effects of intra-articular μHA injection as well as cartilage-on-cartilage tribological characterizations.

Importantly, these results were in striking contrast to those obtained by the authors with geometrically identical PLGA-based microparticles (μPL).^33^ With the μPL, both static and dynamic friction coefficients were observed to increase in a concentration-dependent manner. Unlike μHA, the PLGA μPL lacked a hydrogel structure due to the hydrophobic nature of the polymer and exhibited a much higher stiffness, with a Young’s modulus of ∼5 MPa, which is up to 2 orders of magnitude higher than that of μHA. Also, the rigid PLGA μPL tend to cluster together, increasing resistance during sliding, while the soft μHA deform under tangential loading, generating a more uniform and continuous film at the interface.

Then, given that HA degradation is primarily mediated by reactive oxygen species within the inflamed joint, we investigated the response of these microparticles to a free radical source, specifically H_2_O_2_ in simulated synovial fluid, in the presence of human chondrocytes. Specifically, μHA were exposed to 0.3 mM H_2_O_2_ in simulated synovial fluid, simulating OA conditions,^34^ and their morphology was monitored over the course of 45 days. The μHA size and shape were preserved throughout the entire observation period. This suggests that while H_2_O_2_ interacts with the particle matrix, the μHA remains structurally stable. This trend was confirmed for both low and high-molecular-weight μHA. Leveraging these results, we examined the protective effects of μHA on cells under oxidative conditions. Given the similar stability for both 10-P_25_ and 50-P_30_ particles, we selected the lower molecular weight μHA to perform *in vitro* characterization. First, we demonstrated that μHA exhibits no cytotoxicity across multiple cell lines, including human chondrocytes and fibroblasts. Furthermore, in human chondrocytes exposed to oxidative stresses (0.3 mM H_2_O_2_), treatment with low-molecular-weight (10-P_25_) μHA significantly reduced cytokine levels, restoring values comparable to those of the untreated controls and confirming the protective role against H_2_O_2_-induced inflammation. These findings were further supported by results from a tridimensional OA model, where μHA treatment effectively preserved the extracellular cartilage matrix and inhibited MMP-13 production in the presence of IL-1β. Interestingly, no clear dose-dependent response was observed in this 3D model, which may be related to the mechanism of action of μHA. In this model, the chondroprotective effects of μHA are likely mediated by their antioxidant properties. It is well established that IL-1β stimulates the production of ROS by chondrocytes, which in turn accelerates extracellular matrix degradation.^46^ In this context, μHA is not internalized by the cells but instead acts extracellularly by scavenging ROS, thereby mitigating the degradation process. Given this mode of action, it is plausible that μHA provides their protective effect once a threshold level of ROS neutralization is achieved, beyond which additional doses confer limited incremental benefit. Collectively, these data highlight the antioxidant and anti-inflammatory properties of μHA, reinforcing their therapeutic potential in OA by mitigating ROS-induced damage and inflammation.^47–49^

Finally, a preliminary *in vivo* study was conducted using a murine model of early-stage PTOA. In this experiment, mice were subjected to mechanical loading for 2 weeks and received a single intra-articular injection of low-molecular-weight, high cross-linking μHA (10-P_25_), the clinically used hyaluronic formulation HYALGAN, or saline (control). Since HYALGAN has a molecular weight close to 500 kDa, a 500-P_28_ μHA formulation was specifically prepared for this experiment and directly compared with the clinical product. After 2 weeks, mice were euthanized, and the synovium was isolated to assess the expression of proinflammatory factors. The results indicated that both μHA formulations significantly reduced the elevated expression of IL-1β and TNF-α in the synovium induced by OA, demonstrating superior efficacy compared with the clinical product. The results presented here highlight the translational potential of μHA as a novel intra-articular therapy for OA, driven by both functional and therapeutic advantages. First, the particulate nature of μHA provides a practical advantage in terms of injectability. While currently approved HA hydrogels can be highly viscous and require substantial force to inject, often necessitating large-gauge needles and causing patient discomfort, μHA suspensions can be administered using fine-gauge needles (e.g., 30-gauge, outer diameter of only 310 μm), enabling smoother, less painful injections and potentially improving patient compliance. Typically, viscosupplements are injected using needle gauges ranging from 22 to 18G, corresponding to outer diameters ranging from 720 μm to 1.27 mm. Second, μHA functions as a dual-action therapeutic platform targeting two key aspects of OA pathophysiology: lubrication failure and inflammation. In early OA, cartilage damage triggers inflammation, altering the composition of the synovial fluid and compromising the lubrication layer, where HA serves as the backbone. The resulting increase in friction between the mating interfaces activates chondrocytes, fibroblast-like synoviocytes, and macrophages, leading to the secretion of proinflammatory cytokines (e.g., IL-1β, TNF-α, and others) and catabolic enzymes (MMP-13 and others) that promote cartilage degradation.^50^ The μHA platform is designed to interrupt this degenerative cascade by restoring and maintaining lubrication at both cartilage and synovial interfaces, as suggested by the tribological characterizations in Figure 4, and reducing local inflammatory stimuli, as documented in Figure 7. Additionally, Supplementary Figure 13 further illustrates how μHA localizes to both the cartilage and synovium, where it may provide combined lubricating and anti-inflammatory benefits. Therefore, although we cannot isolate lubrication as the sole mechanism, it is possible that the observed diminished intra-articular inflammation (Figure 8) is partially mediated by improved lubrication. In summary, we propose that the therapeutic efficacy of μHA arises from a dual mechanism: (i) mechanical lubrication and (ii) suppression of inflammation triggered by mechanical and oxidative stress. Beyond these intrinsic effects, μHA particles can be engineered to carry and release bioactive molecules, such as anticatabolic drugs, cytokine inhibitors, growth factors, or gene/RNA therapies, in a controlled, sustained manner. Together, these findings highlight the significant advantages of the μHA platform over current viscosupplements such as HYALGAN (see Figure 8), positioning μHA as a promising next-generation therapy that combines mechanical and pharmacological functions and can be tailored to the disease stage and patients’ needs.

## Conclusions

This study presents a methodology for the fabrication of injectable microscale, cross-linked HA microparticles (μHA) as a dual-functionality system—serving both as a lubricant and anti-inflammatory agent. HA-MA prepolymers, such as 10-P_15_, 10-P_25_ (10 kDa HA with 15% and 25% DM, respectively) and 50-P_17_, 50-P_30_ (50 kDa HA with 17% and 30% DM, respectively) were utilized as photopolymerizable building blocks. Combining a template-based strategy and multistep photopolymerization, μHA were fabricated with precise geometry (20 μm square base, 5 μm height) and tunable mechanical properties by varying the degree of cross-linking and polymer MW. μHA demonstrated lubricant properties, reducing both static and dynamic friction coefficients, and showed resistance to oxidative stress-induced degradation, independent of the molecular weight of the prepolymers. Furthermore, the biocompatibility of 10-P_25_ μHA with various cell types, including chondrocytes and fibroblasts—key components of the joint capsule—was assessed. *In vitro* studies on human chondrocytes showed the ability of10-P_25_ μHA to reduce cytokine levels, protecting against H_2_O_2_-induced inflammation. Furthermore, in a tridimensional OA model, 10-P_25_ μHA preserved cartilage matrix integrity and inhibited MMP-13 production. In a murine OA model, 10-P_25_ μHA significantly reduced the level of synovial expression of IL-1β and TNF-α, outperforming a clinical HA-based product. In sum, these findings highlight the potential of μHA microparticles to improve joint lubrication and modulate molecular processes involved in OA progression, offering a promising mechanopharmacological intervention for this disease. Future studies will aim to optimize the μHA composition to enhance specific adsorption to damaged cartilaginous tissue and enable the delivery of therapeutic agents to halt tissue degeneration and potentially promote regeneration.

## Supplementary Material



## Notes

Synthesis of HA-MA precursors; Preparation and characterization of hyaluronic acid–methacrylate (HA-MA) macroscopic hydrogel; μHA morphological characterization; Tribological apparatus and operation, μHA degradation under extreme oxidative stress conditions; μHA *in vitro* biocompatibility on ATDC5 cells and FLS; list of *p*-values for all the experimental groups of Figures 6 and 8B; Morphology, biocompatibility, and chondroprotective properties of 500-P _28_ μHA; μHA conjugation with Cy5 and Intra-Articular Distribution Analysis (PDF)
