# Supplementary material for: Hyaluronic Acid Microplates for Intra-articular Lubrication and Cartilage Protection in Post-traumatic Osteoarthritis
Source: ACS Appl Mater Interfaces. 2025 Oct 12;17(44):60145–61. doi: 10.1021/acsami.5c11890 (PMC12598699; doi:10.1021/acsami.5c11890)
Supplement: Supplementary file 1 [file am5c11890_si_001.pdf]

SUPPORTING INFORMATION

Hyaluronic Acid MicroPlates for Intra-Articular Lubrication and Cartilage Protection in post-Traumatic Osteoarthritis

Agnese Fragassi [1♣], Antonietta Greco [1♥], Megan Keech[2], Amelia Soltes[2], Fang Yu[2], Sayanti Brahmachari[1], Roberto Palomba[1], Martina di Francesco[1], Miguel Echanove Gonzalez De Anleo[3], Froilan Granero-Molto[3], Luca Ceseracciu[4], Veronica Papa[1], Luca Goldoni[4], Aiman Abu Ammar[6], Richard D'Arcy[2♦], Haytam Kasem[5] Craig Duvall[2□], and Paolo Decuzzi[1,7□]

[1] Laboratory of Nanotechnology for Precision Medicine, Fondazione Istituto Italiano di Tecnologia, Via Morego 30, Genova 16163, Italy

[2] Department of Biomedical Engineering, Vanderbilt University, 2301 Vanderbilt Place, Nashville, TN 37235-1826, United States

[3] Cell Therapy Area, Clínica Universidad de Navarra, Av. de Pío XII, 36, 31008 Pamplona, Navarra, Spain

[4] Materials Characterization Facility, Istituto Italiano di Tecnologia, Via Morego 30, Genova 16163, Italy

[5] Biotribology Inter-Disciplinary Research Center, Azrieli College of Engineering Jerusalem, 26 Yaakov Shreibom Street, Ramat Beit Hakerem, Jerusalem 9103501, Israel

[6] Department of Pharmaceutical Engineering, Azrieli College of Engineering Jerusalem, Jerusalem 9103501, Israel

[7] Department of Medicine, Division of Oncology, Stanford University School of Medicine, 269 Campus Drive, Stanford, CA 94305, USA

♣ Present Address: Department of Pharmaceutical and Pharmacological Sciences, University of Padova, Via F. Marzolo 5, 35131 Padova, Italy.

♥ Present Address: Department of Medicine and Surgery, NanoMedicine Center (NANOMIB), University of Milano-Bicocca, Via Follereau 3, 20854 Vedano al Lambro, Italy

♦ Present Address: Chemical Engineering, School of Engineering of Matter, Transport and Energy, Arizona State University, Tempe, AZ, USA

□ Shared senior authorship

Corresponding author: paolo.decuzzi@iit.it

Supplementary Methods and Results

A

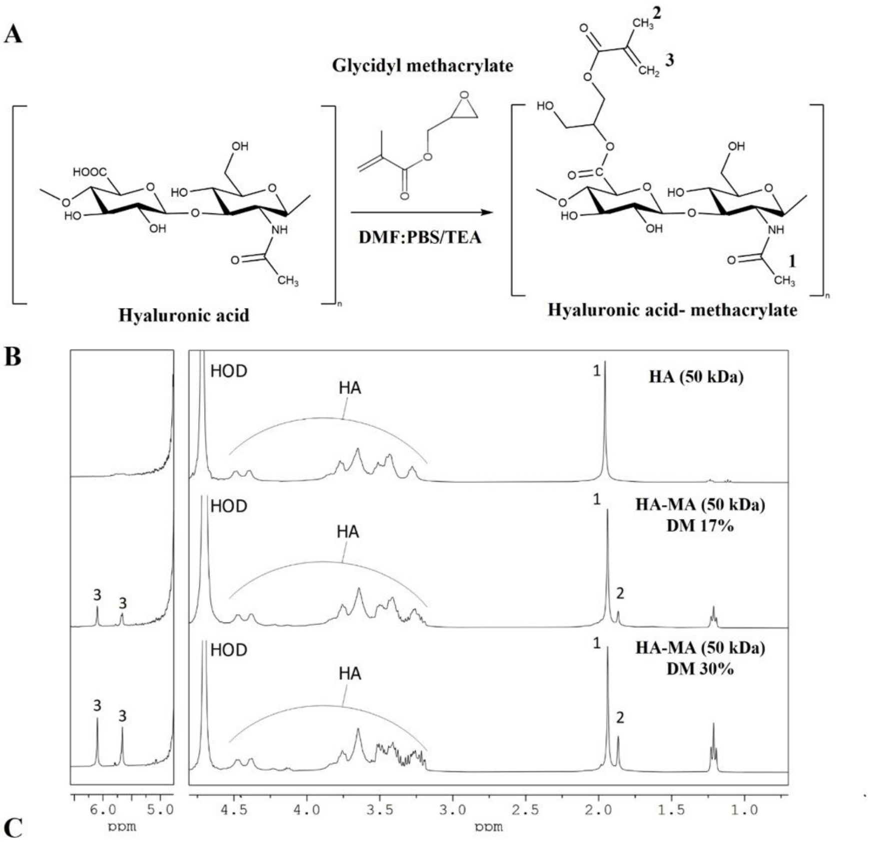

C

HA 10 kDa

HA/GM	Time (days)	DM	HA-MA
1/50	0.5	11	10-P_{11}
**1/50**	**2**	**15**	**10-P_{15}**
1/50	5	19	10-P_{19}
1/100	0.5	15	10-P_{11}
1/100	2	16	10-P_{16}
**1/100**	**5**	**25**	**10-P_{25}**

HA 50 kDa

HA/GM	Time (days)	DM	HA-MA
1/50	0.5	14	50-P_{14}
**1/50**	**2**	**17**	**50-P_{17}**
1/50	5	19	50-P_{19}
1/100	0.5	15	50-P_{15}
1/100	2	19	50-P_{19}
**1/100**	**5**	**30**	**50-P_{30}**

Supplementary Figure 1. Synthesis of HA-MA precursors. A. Methacrylation reaction of HA with GM. **B**. NMR of HA (50 kDa) and HA-MA with different DM (17 and 30%). **C.** HA-MA pre-polymers synthesized by varying the HA/GM ratio (50 or 100-fold) and the reaction time (12 hours, 2 or 5 days). The HA-MA in orange for 50 kDa and blue for 10 kDa were selected as building block to generate microparticles.

Preparation and characterizations of hyaluronic acid–methacrylate (HA-MA) macroscopic hydrogel. HA-MA pre-polymers were assembled into macroscopic hydrogels (macrogels) via photo-polymerization. To this end, HA-MA precursors were dissolved at varying weight percentages (2.5, 5, 10, 15, 20, 25, 30% w/v) in deionized water with 0.1% w/v photoinitiator Lithium phenyl (2,4,6-trimethylbenzoyl) phosphinate (LAP). This solution (200 µL) was placed in a mold with square wells and exposed to UV-light (365 nm, 10 mW cm^{-2}) for 10 minutes. Note that the conditions for hydrogel formation for each pre-polymers were determined by exposing progressively concentrated solutions (from 2.5 to 30% w/v) of HA-MA to UV light for 5 minutes and verifying the occurrence of the sol-gel transition. Suitable polymerization conditions ranged between the HA-MA concentrations for critical gelation concentration (CGC) and the solubility limit (SL) in water, as indicated by the purple bars in **Supplementary Figure 2A**. Data showed that the 10 kDa HA-MA precursors exhibited a broader suitable polymerization window as compared to the longer 50 kDa HA-MA precursors, which, as expected, displayed a lower critical gelation concentration (**Supplementary Figure 2C**).

To evaluate the swelling behavior, HA-MA macroscopic hydrogels at fixed pre-polymer concentration (HA-MA 10% w/v) were weighted and placed in deionized water. After 24 hours, the water was completely removed using a pipette, and the hydrogels were left to dry overnight on a 0.2 µm filter. Then, HA-MA hydrogels were weighted again to determine the amount of adsorbed water. The swelling ratio was calculated by dividing the swollen gel mass by the dried gel mass.

The results showed that an increase in DM led to a statistically significant decrease in the swelling ratio for the longer (50 kDa) HA chain hydrogels, whereas no significant difference was observed for the shorter (10 kDa) HA chain hydrogels (**Supplementary Figure 3A**). Interestingly, the polymer chain length did not significantly impact the swelling trend, as the swelling ratio of hydrogels of 10-P_{25} was lower than that of hydrogels of 50-P_{17}. In particular, the swelling ratio of the hydrogels decreased from 3.6 ± 0.3 (10-P_{15}) to ~3.2 ± 0.1 (10-P_{25}), for the for 10 kDa HA-MA; and from 4.1 ± 0.1 (50-P_{17}) to 2.2 ± 0. (50-P_{30}), for the for 50 kDa HA-MA.

The stiffness of the hydrogels was assessed through uniaxial unconfined compression conducted on an Instron 3365 dynamometer equipped with a 10 N load cell at the constant rate of 1 mm/min. Swollen hydrogels prepared with a fixed pre-polymer concentration (HA-MA 10% w/v) were tested ex-liquid at 12 hours post-hydration, removing excess water just before loading on the instrument. The elastic modulus was determined as the slope of the stress-strain curve at 12-15%

strain. In **Supplementary Figure 3B**, the representative stress-strain curves for hydrogels formed with the two extremes precursors, namely 10-P_{15} and 50-P_{30}, are depicted. For each hydrogel, the general slope was quasi linear at low strains (< 0.10). The elastic Young's modulus was determined as the slope of the linear region stress-strain curve up to 10% strain. As illustrated in **Supplementary Figure 3C**, a statistically significant increase in Young's modulus was observed with increasing DM for both MW. Additionally, hydrogels with higher molecular weight also exhibited greater stiffness. Specifically, the elastic modulus of the hydrogels increased from 19 ± 3.10 kPa (10-P_{15}) to 59.1 ± 2.8 kPa (10-P_{25}), for the for 10 kDa HA-MA; and from 71.3 ± 11.9 kPa (50-P_{17}) to 125.3 ± 20.2 kPa (50-P_{30}), for the for 50 kDa HA-MA.

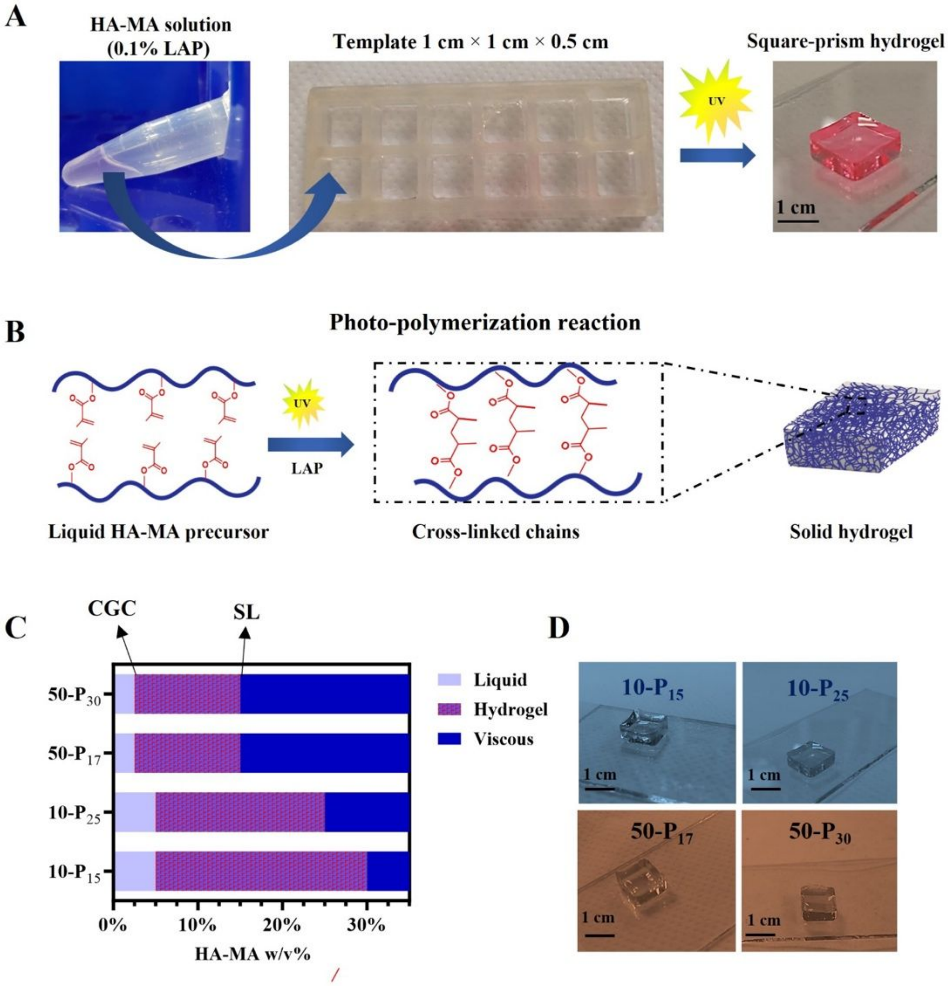

Supplementary Figure 2. Macroscopic hydrogel production. **A**. Pictures of the initial HA-MA solution with 0.1% LAP, the macroscale hydrogel template with square wells used to form hydrogel blocks and the square prism-hydrogel obtained after exposure to UV light. **B**. Photo-

polymerization reaction and cross-linked network formation after exposure of the HA-MA solution to UV light. **C**. Chart for the hydrogel formation window vs. pre-polymer concentration for the HA-MA precursors (CGM: critical gel concentration, SL: solubility limited). **D.** Pictures of the hydrogel blocks derived from the four HA-MA pre-polymers as starting material.

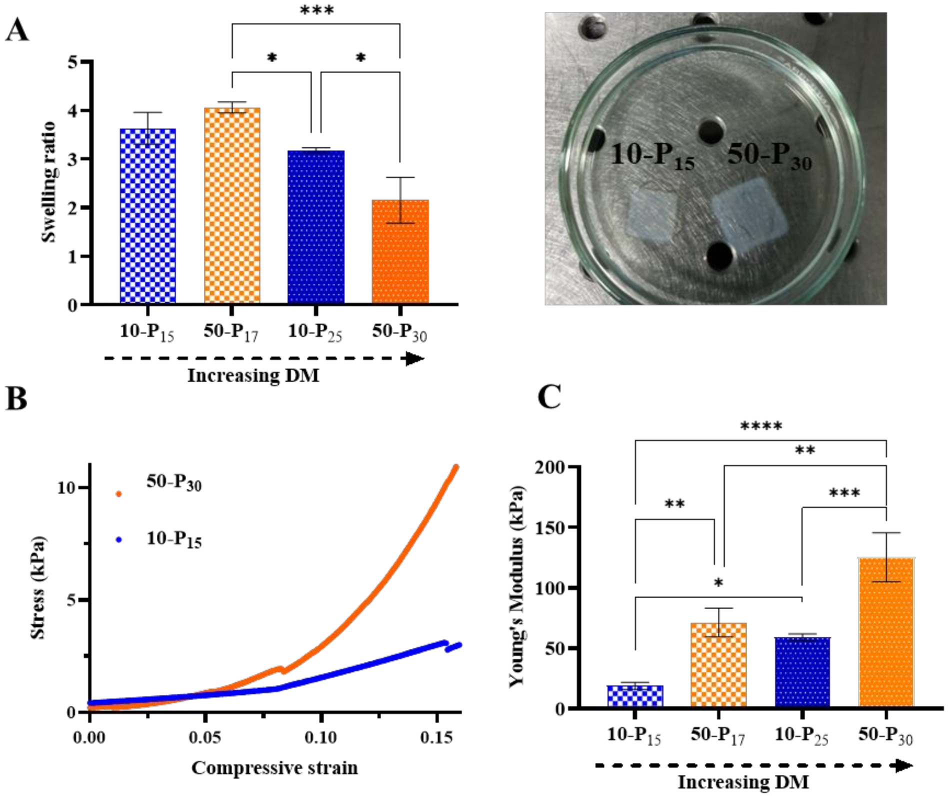

Supplementary Figure 3. Macroscopic hydrogel characterization. **A**. Influence of the DM and MW on the swelling ratio of hydrogels formed using 10% w/v as pre-polymer concentration. On the right picture of 50-P_{17} and 50-P_{30} hydrogels after reaching swelling equilibrium. **B**. Representative stress–strain curves of macroscopic hydrogels of 10-P_{15} and 50-P_{30} generated during uni-axial compression. **C**. Stiffness of hydrogels formed using 10% w/V as pre-polymer concentration. Results are presented as mean ± SD (N= 3). Statistical analysis via one-way ANOVA (GrapHAad Prism 10): * indicates $p < 0.05$, ** indicates $p< 0.01$, *** indicates $p < 0.001$ and **** indicates $p<0.0001$. No statistically significant differences are not indicated on the graphs.

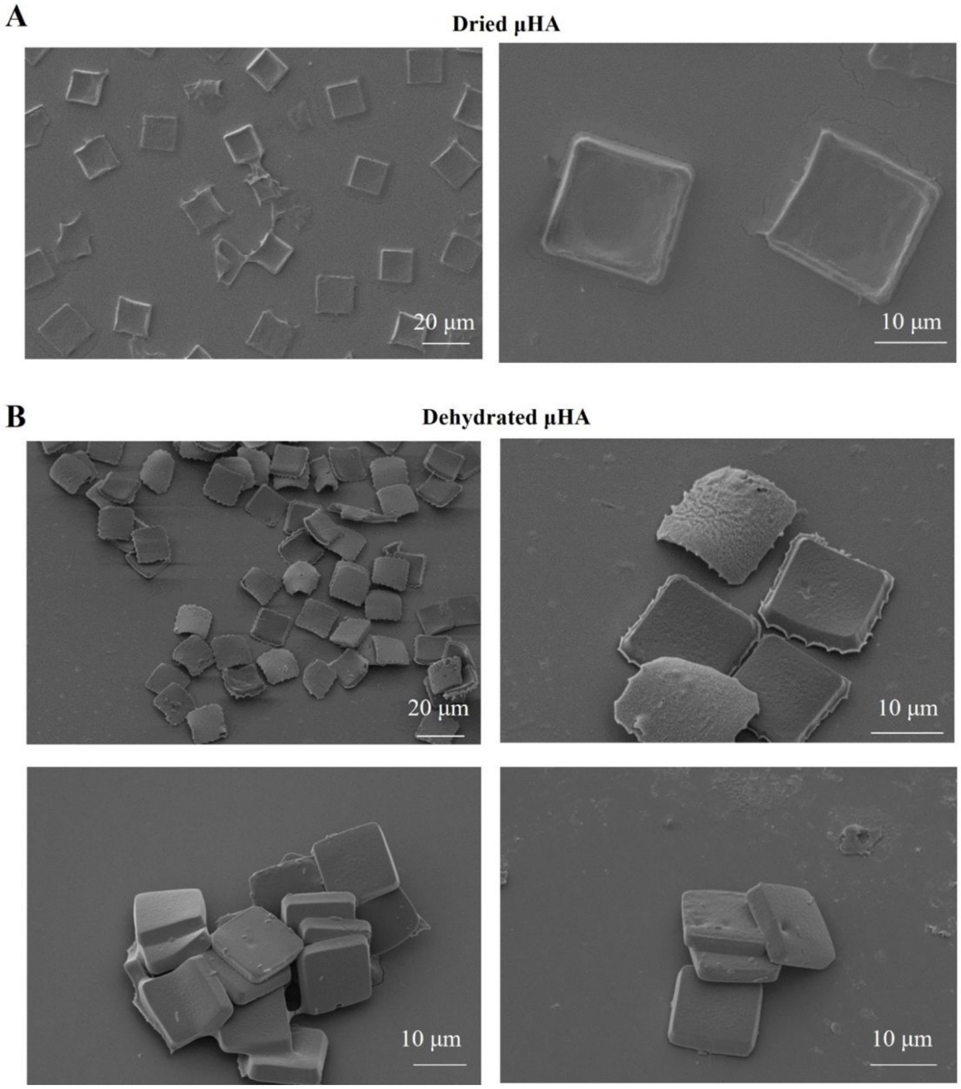

Supplementary Figure 4. µHA morphological characterization. **A**. SEM pictures of dried µHA produced using 10-P_{25}. **B.** SEM pictures of µHA dehydrated with Ethanol. On the top pictures are related to µHA produced using 10-P_{25} as building block. On the bottom pictures are related to µHA produced using 50-P_{30} as building block.

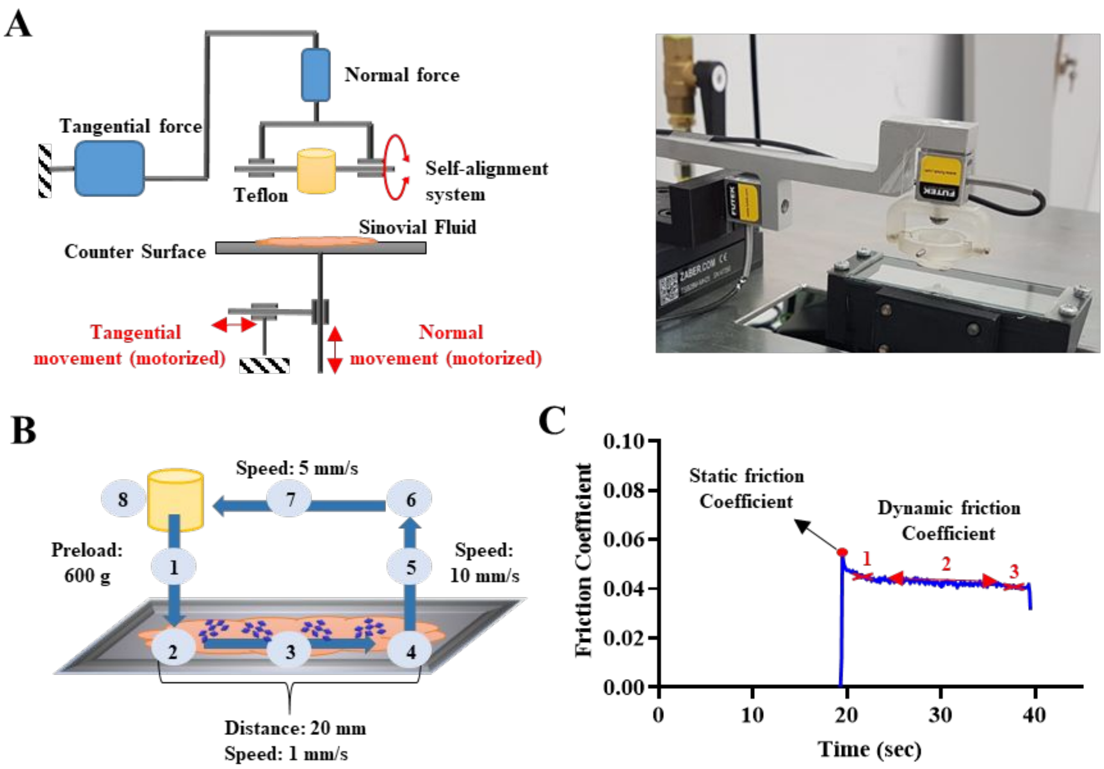

Supplementary Figure 5. Tribological apparatus and operation. A. Schematic representations (left) and image (right) of a customized linear two-axis tribometer used for the tribological characterizations. **B**. The apparatus is operated following a cycle of 8 consecutive steps, including the sliding phase 3 during which tangential forces are continuously measured under a fixe normal load (5.8 N). **C.** A representative curve showing the typical variation of the friction coefficient during the sliding step. The static coefficient of friction was calculated at the onset of sliding (red dot), whereas the dynamic coefficient of friction was calculated by averaging the tangential force during the stable sliding phase (red double edge arrow – {2}).

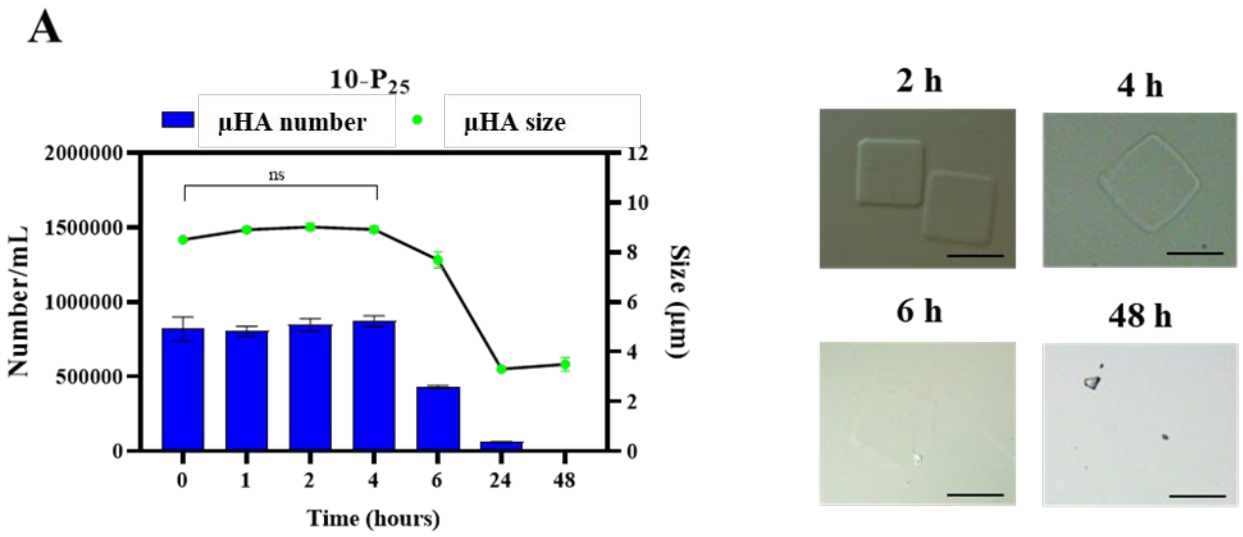

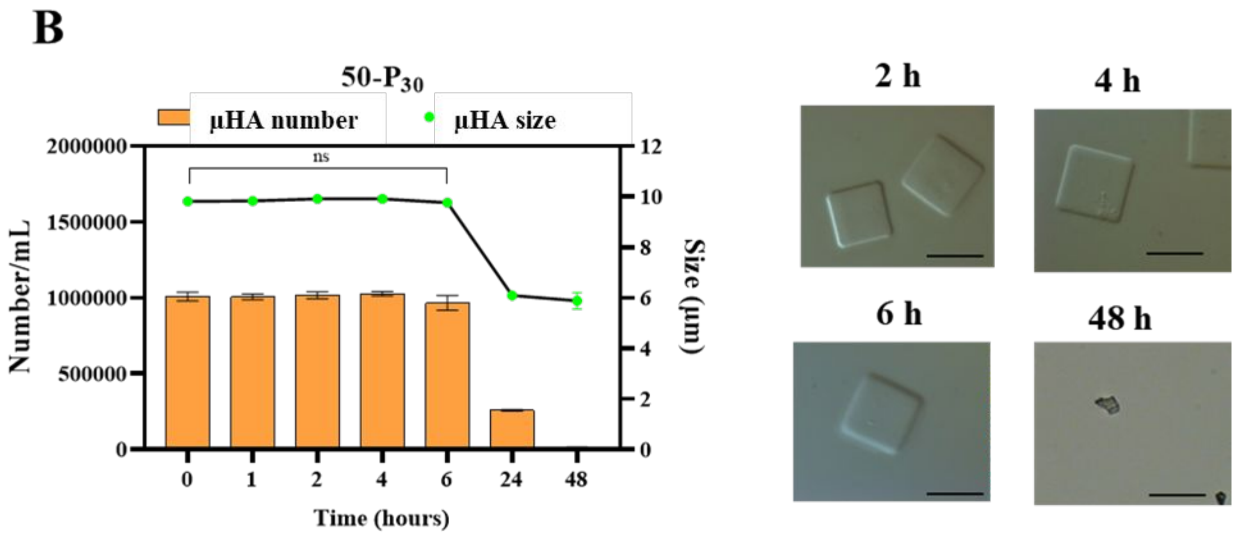

Supplementary Figure 6. µHA degradation in extreme oxidative stress conditions. **A.** Size analysis via Multisizer and microscopy analysis of 10-P_{25} µHA incubated in simulated synovial fluid with H_2O_2 at different predetermined time points. **B.** Size analysis via Multisizer and microscopy analysis of 50-P_{30} µHA incubated in simulated synovial fluid with H_2O_2 at different predetermined time points. The statistical analysis was performed by two-way ANOVA (GrapHAad Prism 10) with Tukey's test. All the results are mean values ± SD of three independent experiments (Scale bar: 20 µm).

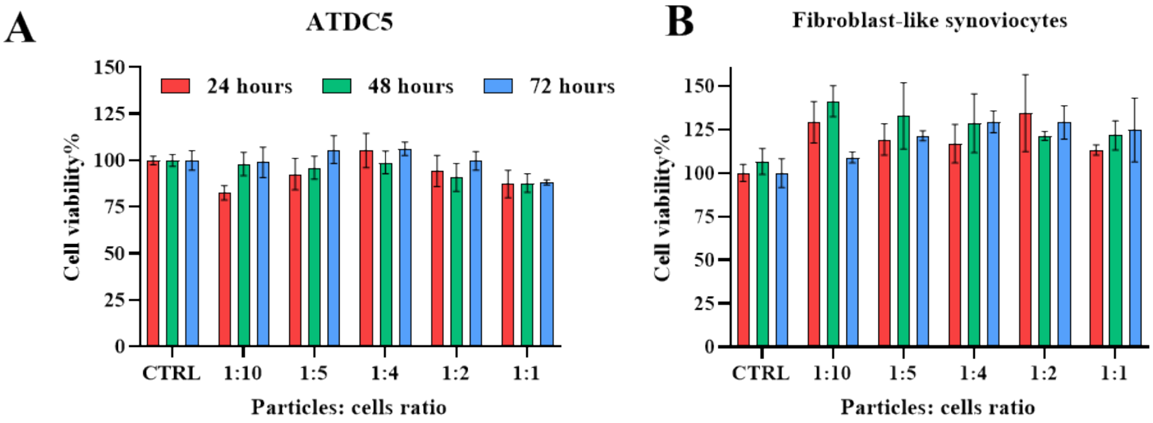

Supplementary Figure 7. µHA *in vitro* biocompatibility. A. Viability of human ATDC5 cells upon incubation with different amounts of 10-P_{25} µHA, for 24, 48 and 72 hours. **B**. Viability of FLS upon incubation with different amounts of 10-P_{25} µHA, for 24, 48 and 72 hours.

Cytokine	Groups	Adjusted P-value
IL-1β	CTRL vs. No treatment	<0,0001
	CTRL vs. 10-P_{25} µHA	0,0004
	No treatment vs. 10-P_{25} µHA	0,0015
TNF-α	CTRL vs. No treatment	<0,0001
	CTRL vs. 10-P_{25} µHA	0,0448
	No treatment vs. 10-P_{25} µHA	<0,0001
IL-6	CTRL vs. No treatment	<0,0001
	CTRL vs. 10-P_{25} µHA	0,0047
	No treatment vs. 10-P_{25} µHA	<0,0001

Supplementary Figure 8. Statistically significant differences for all the experimental groups of **Figure 6B**. Significance corresponds to a p-value smaller than 0.05.

Two-stage linear step-up procedure of Benjamini. Krieger and Yekutieli	Individual P -Value	
	**IL-1β**	**TNF-α**
SHAM vs. SALINE	0.0019	0.0104
SHAM vs. HYALGAN	0.0065	0.0096
SHAM vs. 10-P_{25}-μHA	0.1466	0.5370
SHAM vs. 500-P_{28}-μHA	0.1943	0.6463
SALINE vs. HYALGAN	0.5718	0.9674
SALINE vs. 10-P_{25}-μHA	0.0346	0.0320
SALINE vs. 500-P_{28}-μHA	0.0335	0.0160
HYALGAN vs. 10-P_{25}-μHA	0.1075	0.0294
HYALGAN vs. 500-P_{28}-μHA	0.0993	0.0146
10-P_{25} μHA vs. 500-P_{28}-μHA	0.9049	0.8443

Supplementary Figure 9. Statistically significant differences for all the experimental groups of **Figure 8B**. Significance corresponds to a p-value smaller than 0.05.

Morphology, biocompatibility, and chondroprotective properties of 500-P_{28} μHA. Given that the commercially available product HYALGAN has a molecular weight of approximatively 500 kDa, we fabricated 500 kDa μHA using the same protocols and conditions as those employed for the synthesis of 50-P_{25} kDa μHA. Specifically, 500 kDa HA-MA precursors with two distinct DM (18 and 28%) were synthesized following the procedure described in the **Methods** (**Supplementary Figure 10A**). Due to the lower solubility of these polymers, macrogels were formed at a pre-polymer concentration of 7.5% w/v. The stiffness of these two hydrogels was found to be approximately □100 – 150 kPa, with no statistically significant differences between the two precursors (**Supplementary Figure 10B**). To enable a direct comparison with the 10-P_{25} HA-MA, we selected the 500-P_{28} precursor to generate microparticles. **Supplementary Figure 10C** illustrates the size distribution of the microparticles produced using a 7.5% w/v pre-polymer concentration and the same multi-step polymerization process detailed in the **Methods**. Concentrations of 7.5% w/v of HA-MA generated batches of μHA with narrow size distribution and yielding values comparable to those obtained with the other precursors. The mechanical properties of these microparticles were evaluated using nanoindentation and damping analyses, showing a Young's modulus comparable to that of the bulk hydrogel (□142 kPa, **Supplementary Figure 10D**). Additionally, the degradation profile of the microparticles was assessed under OA-like and extreme oxidative conditions. Data showed that these microparticles resist degradation by H_2O_2 for up to 35 days under OA-like conditions (**Supplementary Figure 11A**, **B**) and for 24 hours under extreme oxidative conditions (**Supplementary Figure 11C**).

Moreover, ATDC5-derived chondrocyte spheroids, which form tri-dimensional cartilage aggregates, were used to examine their chondroprotective properties. To replicate the inflammatory environment associated with OA, ATDC5-based tridimensional cartilage aggregates on day 28 were exposed to IL-1β (5 ng/mL) for 72 hours, with or without μHA. At the end of the treatment, cell media were collected to assess the extent of matrix degradation, measuring GAG content in the media to assess GAG release/degradation and determining MMP-13 activity as markers of collagen breakdown. Media analysis demonstrated that these HA formulations provided protective effects against IL-1β – induced matrix degradation. Notably, the presence of

μHA reduced GAG release by approximately 30% and suppressed MMP-13 activity in the media by 90% for the highest amount of microparticles. While the chondroprotective effect of 10-P_{25} μHA did not consistently correlate with HA concentration, the 500-P_{28} HA-μHA formulation demonstrated a much stronger, dose dependent reduction in MMP-13 activity (**Supplementary Figure 12**).

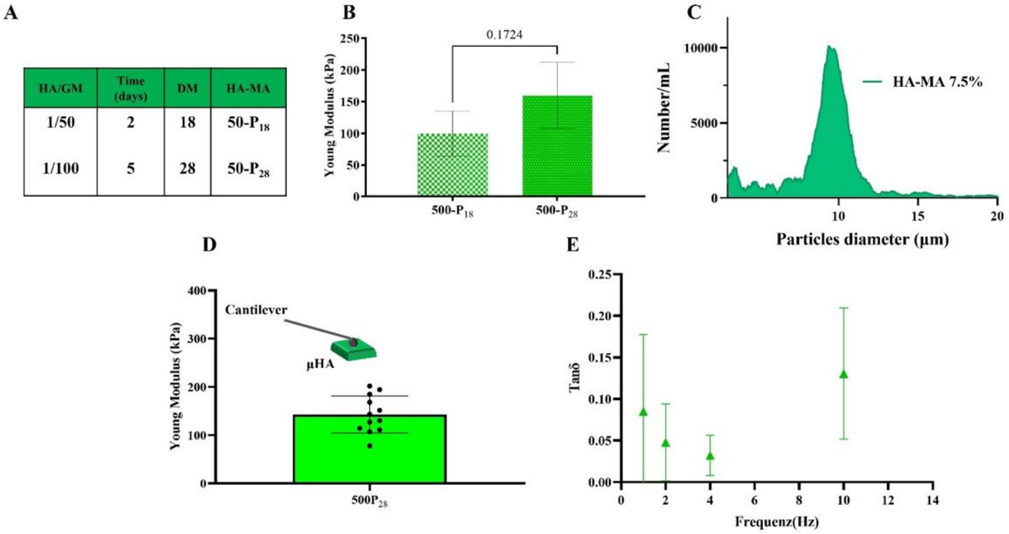

Supplementary Figure 10. 500 kDa HA-MA characterization as bulk hydrogel and μHA. A. HA-MA (500 kDa) pre-polymers synthesized by varying the HA/GM ratio (50 or 100-fold) and the reaction time (2 or 5 days). **B**. Stiffness of hydrogels (500-P_{18} and 500-P_{28}) formed using 7.5 % w/V as pre-polymer concentration. **C**. Size distribution of μHA fabricated using the HA-MA (500-P_{28}) at different % w/v concentrations. **D.** Apparent Young's modulus for 500-P_{28} μHA. **E.** Mechanical damping of 500-P_{28} μHA upon cyclic loading as a function of the frequency.

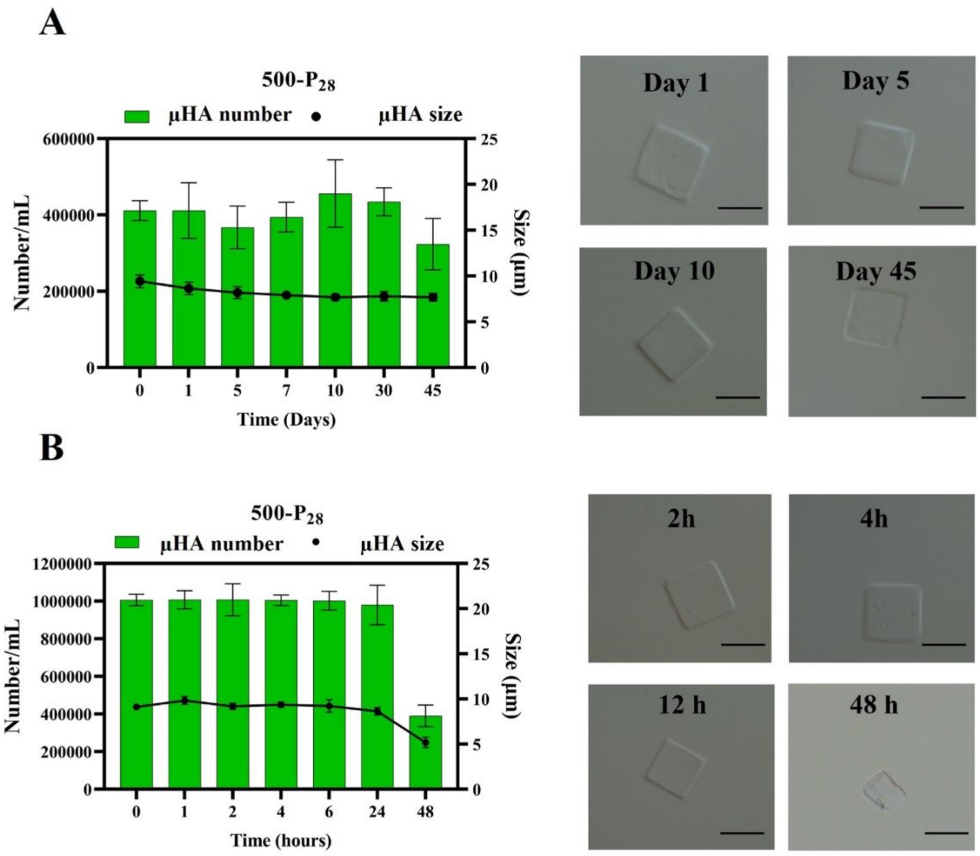

Supplementary Figure 11. 500-P_{28} μHA degradation under oxidative stress conditions. A. Size distribution analysis via Multisizer and Microscopy analysis of 500-P_{28} μHA at predetermined time point during incubation with 0.3 mM H_2O_2 in simulated SF. **B**. Size distribution analysis via Multisizer and Microscopy analysis of 500-P_{28} μHA at predetermined time point during incubation with pure H_2O_2 (Scale bar: 20 μm).

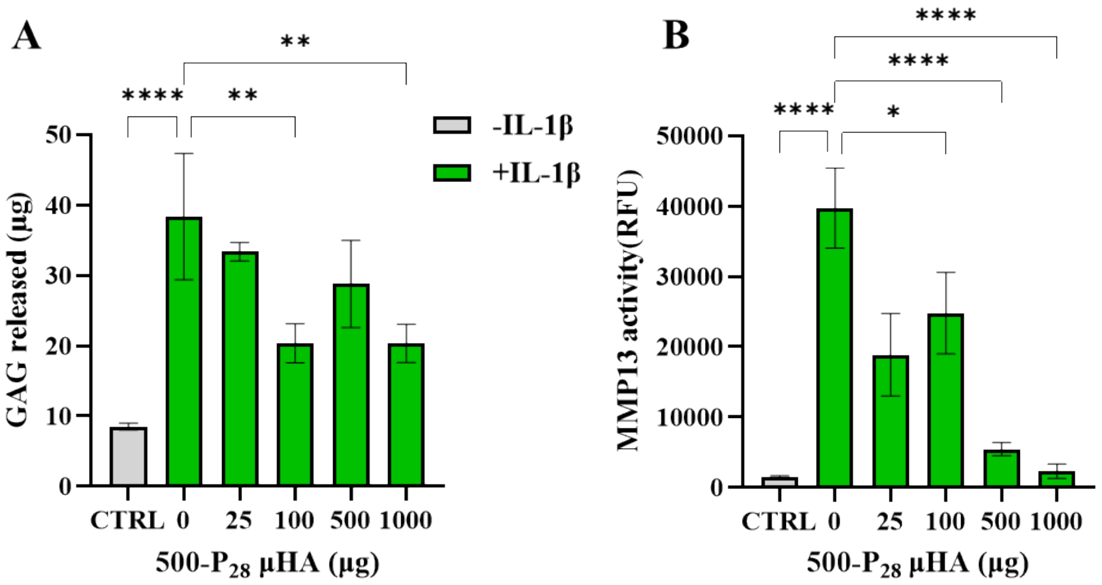

Supplementary Figure 12. **500-P_{28} µHA chondroprotective effect on ATDC5 aggregates stimulated with IL-1β. A.** GAG released in the media for ATDC5 tridimensional aggregates stimulated with IL-1β for 72 h and exposed to different concentrations of 500 kDa µHA. **B.** MMP13 activity in the media for ATDC5 tridimensional aggregates stimulated with IL-1β for 72 h and exposed to different concentrations of 500 kDa µHA. The CTRL (gray bar) is related to ATDC5 tridimensional aggregates that were not stimulated with IL-1β. Results are presented as mean ± SD (n = 3). Statistical analysis via one-way ANOVA (Graphad Prism 10): * indicates $p < 0.05$, ** indicates $p< 0.01$, *** indicates $p < 0.001$ and **** indicates $p<0.0001$. "No significance" is not indicated on the graphs.

μHA Conjugation with Cy5 and Intra-Articular Distribution Analysis

To evaluate μHA distribution within the joint, Cy5 was covalently attached to the particle surface, ensuring stable fluorescent labeling. Briefly, 1 mg of lyophilized μHA was reconstituted in 100 μL of 0.1 M HEPES buffer (pH 7.4), containing approximately 2.5 mmol of carboxylate groups. To this solution, 24 μL of a 1 mg/mL Cy5-NH_2 solution in DMSO (0.325 μmol; targeting ~1% carboxylate substitution, 1.3 equivalents) was added. This was followed by 45 μL of a 10 mg/mL DMTMM solution in HEPES (1.625 mmol; 0.65 equivalents) to activate the conjugation reaction. After 24 hours of incubation, excess unbound Cy5 was removed through repeated centrifugation at 2500 g for 5 minutes. Successful Cy5 conjugation to the μHA was confirmed using confocal microscopy (Nikon Eclipse Ti inverted confocal microscope).

To assess intra-articular distribution, Cy5-labeled μHA (10 mg/mL, synthesized using the 10-P25 building block) was administered via a single intra-articular injection in mice (n = 5) with mechanically induced post-traumatic osteoarthritis (PTOA). Following euthanasia, the legs were stored at −80 °C, embedded in OCT compound, and serially sectioned in the sagittal plane until the joint space was adequately exposed. Cryosections (20 μm thick) were collected at various depths using a polyvinylidene chloride film coated with synthetic rubber cement and mounted on slides. Sections were fixed in 10% neutral buffered formalin for 5 minutes, coverslipped with Aqua-Mount, and imaged using confocal microscopy.

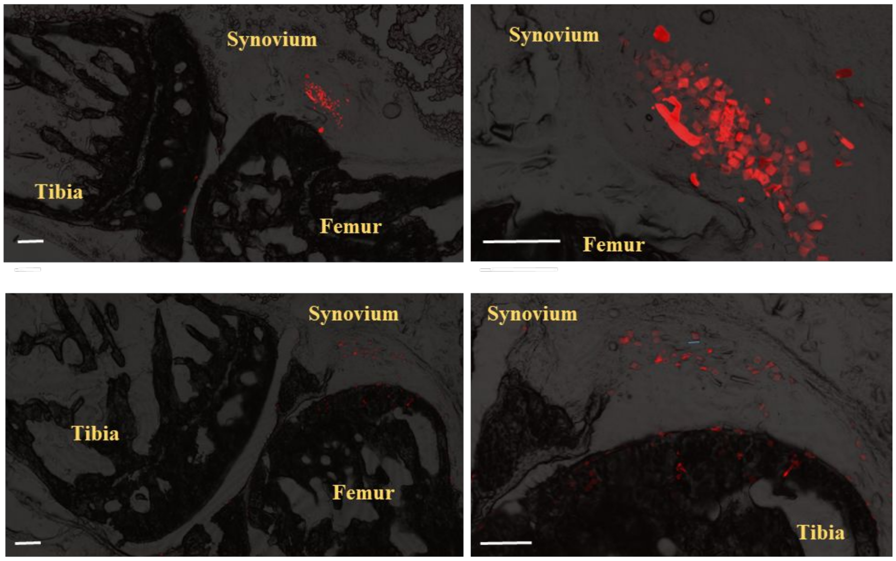

Supplememtary Figure 13. Cryo-sectioning and confocal microscopy. Confocal microscopy imaging performed on cryosections take from tissues harvested 1 day after intra-articular injection. Imaging showed Cy5-µHA located on top of the cartilage surface, near the cartilage/synovium interface, and along the joint capsule (scale bar = 100 µm).
